# Is age associated with different vital signs in adults presenting to hospital with bacterial infection? A systematic review and meta-analysis

**DOI:** 10.1093/ageing/afaf194

**Published:** 2025-07-23

**Authors:** Phoebe Tupper, Oliver Redfern, Charlotte H Harrison, Stephen Gerry, Christopher Biggs, Bethany Walker, Peter Watkinson

**Affiliations:** Critical Care Research Group, Kadoorie Centre for Critical Care Research and Education, Nuffield Department of Clinical Neurosciences, University of Oxford, Oxford, UK; Critical Care Research Group, Kadoorie Centre for Critical Care Research and Education, Nuffield Department of Clinical Neurosciences, University of Oxford, Oxford, UK; Critical Care Research Group, Kadoorie Centre for Critical Care Research and Education, Nuffield Department of Clinical Neurosciences, University of Oxford, Oxford, UK; Centre for Statistics in Medicine, Nuffield Department of Orthopaedics, Rheumatology and Musculoskeletal Sciences, University of Oxford, Oxford, UK; Critical Care Research Group, Kadoorie Centre for Critical Care Research and Education, Nuffield Department of Clinical Neurosciences, University of Oxford, Oxford, UK; Critical Care Research Group, Kadoorie Centre for Critical Care Research and Education, Nuffield Department of Clinical Neurosciences, University of Oxford, Oxford, UK; Critical Care Research Group, Kadoorie Centre for Critical Care Research and Education, Nuffield Department of Clinical Neurosciences, University of Oxford, Oxford, UK; Oxford Critical Care, Oxford University Hospitals NHS Trust, Oxford, UK; Oxford Biomedical Research Centre, National Institute for Health and Care Research (NIHR), Oxford, Oxfordshire, UK

**Keywords:** infection, age, vital signs, diagnosis, systematic review, older people

## Abstract

**Background:**

It has long been suspected that the vital sign abnormalities that accompany bacterial infection are subtle or absent in older adults. This review summarises the evidence for whether older adults present with different vital sign abnormalities to younger adults when hospitalised with bacterial infection.

**Methods:**

MEDLINE, EMBASE and CINAHL EBSCO were searched from inception to 19 December 2024 for English-language research articles of patients hospitalised with bacterial infection reporting age and admission vital signs. We used meta-regression to assess how vital signs vary with age. Where studies reported vital signs in multiple age groups, we undertook a meta-analysis in younger (<65) and older patients (≥65). Evidence quality was assessed using an adapted Quality Assessment of Diagnostic Accuracy Studies-2 tool.

**Results:**

Our search yielded 14 487 studies; 132 were included after screening. Older adults were less likely to be tachycardic (RR 0.82, 0.69 to 0.97, *I*^2^ = 86.5%) with a mean difference in heart rate of 5 bpm (−7 to −3 bpm, *I*^2^ = 88.3%). Older adults were less likely to be febrile (RR 0.89, 0.83 to 0.95, *I*^2^ = 85.9%) with a mean difference in temperature of 0.14°C (−0.26 to −0.02°C, *I*^2^ = 94.6%). Most (129/132) studies were at high risk of bias.

**Conclusions:**

Whilst differences in absolute values were small, there was consistency in the finding that older adults were less likely than younger adults to be tachycardic or febrile. As vital signs at presentation may prompt suspicion of infection, influencing investigations and treatment, special consideration for the possibility of infection in older patients with normal vital signs may be warranted.

## Key Points

- Patient age is associated with significant differences in the vital signs that accompany infection.
- Older adults with bacterial infection are less likely to be tachycardic or febrile.
- Older adults with bacterial infection are more likely to be tachypnoeic.

## Introduction

The population aged over 65 is growing faster than any other age group [1]. Infection disproportionately affects older adults [2, 3]. Explanations for this include age-related changes to the immune system; comorbidities and medications causing immunosuppression; and weakened physical barriers to infection (e.g. neurological disease increasing the risk of aspiration) [4, 5].

Vital signs are objective measures of patient physiology. They include temperature, heart rate (HR), systolic blood pressure (SBP), respiratory rate (RR), oxygen saturation (SaO2) and level of consciousness. Early warning scores amalgamate vital signs into a single score, to aid recognition of deteriorating patients and standardise communication between healthcare professionals [6, 7]. Vital signs have also been incorporated into screening tools to help identify sepsis [8].

Infection in older adults leads to worse outcomes, including higher mortality. Reasons are multifactorial, but late diagnosis and initiation of treatment, due to atypical presentation, may be partly responsible [4, 9–11]. It has long been suggested that older patients exhibit a blunted host response to infection, contributing to atypical symptoms and signs [12]. In 1892 William Osler observed, ‘in old age pneumonia may be latent, coming on without chill; the cough and expectoration are slight, the physical signs ill-defined and changeable, and the constitutional symptoms out of all proportion’ [13]. It is possible that this extends to vital signs with the National Institute for Health and Care Excellence (NICE). Sepsis guidelines advising that older patients may not present with fever or a raised HR [14]. However, the quality of evidence on which this statement was based was deemed ‘very low’ and some studies suggest that the presentation of infection in older patients may not be as ‘atypical’ as traditionally believed [12].

It is vital that clinicians understand the presentation of infection in older adults. If older adults present with fewer vital sign abnormalities, there may be a delay in recognition of infection. One study showed that the ratio of completed 1-h sepsis bundles was significantly higher amongst patients with an increased body temperature, suggesting fever was associated with a higher quality of care [15]. This is important as rapid initiation of appropriate treatment has been shown to improve outcomes [16–18]. Conversely, overdiagnosis of infection, something studies have suggested is frequent in the emergency department (ED), may lead to unnecessary investigation and treatment [16, 19, 20]. Treatment may include broad-spectrum antibiotics, which contributes to antimicrobial resistance and adverse drug effects (of particular concern in a population with high incidence of renal failure and polypharmacy) [5].

This systematic review summarises whether age is associated with different vital sign abnormalities when adults present to hospital with bacterial infection. Whilst several narrative reviews have discussed the presentation of infection in older patients, we are unaware of any systematic review or meta-analysis that combines or critically appraises the available evidence [21–26].

## Materials and methods

This study is registered on PROSPERO, number CRD42021298756, where the protocol can be accessed.

### Inclusion criteria

Study type: We included primary research articles describing admission vital signs in patients hospitalised with a diagnosis of bacterial infection. We included cross-sectional, cohort, case–control studies and randomised controlled trials. Case reports, case series and conference abstracts were excluded. Systematic and narrative reviews were excluded but, where relevant, their references were used for identification of primary literature.

Setting: We included studies of patients attending hospital; *via* the ED or direct admission under medical or surgical teams. Studies of patients who did not attend hospital were excluded.

Population: We included patients with proven or suspected bacterial infection. This included bacteraemia, pneumonia (excluding infective exacerbations of COPD and asthma), urinary tract infections, skin and soft tissue infections, intra-abdominal infections, bacterial meningitis and bacterial infective endocarditis. ‘Bacterial infection’ was defined at the study level—this could be based on specific culture results, clinical criteria (based on symptoms, imaging or investigation results) or clinical consensus. We excluded studies of viral, fungal or parasitic infections. Studies including patients who were pregnant or below the age of 18 years were excluded. We also excluded studies in immunosuppressed participants and those that selected patients with a common comorbidity.

Exposure and control: We wanted to examine the association of patient age with presenting vital signs. Therefore, for inclusion, studies must have either had inclusion criteria defined by a specific age range (e.g. patients aged over 75 years) or have performed age-stratified analysis of vital signs, enabling data for patients of different age groups to be compared. Studies reporting vital signs for just one age group were eligible for inclusion in the meta-regression analysis looking at the relationship between continuous age and vital signs. Studies presenting data on two or more age groups were eligible for inclusion in the dichotomous meta-analysis where vital signs were compared between older and younger age groups.

Outcomes: We included studies that had measured HR, RR, blood pressure, SaO2 or temperature. We included studies that reported vital signs in continuous or dichotomous (e.g. tachycardia, fever) format. For continuous vital signs, we extracted the average measure and a measure of variability. For dichotomous vital signs, we extracted the threshold used, the number of patients falling into each category and the total number of patients. A full list of outcomes extracted can be found in supplementary material (Appendix E).

Vital signs measured using continuous or ambulatory monitoring were excluded. We included studies that described vital signs taken within 24 h of admission to minimise the impact of medical treatment on vital signs. Admission also represents a key point at which decisions are made regarding diagnosis.

### Information sources

The MEDLINE Ovid (including epub ahead of print and in-process and other non-indexed citations, 1946 to present), EMBASE Ovid (1974 to present) and CINAHL EBSCO (1982 to present) databases were searched from inception to 6 September 2021. Update searches on 14 June 2023 and 19 December 2024 identified articles published since the original search. Search strategies included MeSH terms and free text and were developed for each database by clinicians working alongside a research librarian. The search was limited to studies published in the English language. The full search strategies can be found in the supplementary material (Appendix B). Forward and backward citation tracking of included papers using Web of Science, was done on the day each paper was reviewed for inclusion.

#### Selection process and data collection

All identified references were downloaded to a reference library software (Covidence). After removal of duplicates, two reviewers independently reviewed titles and abstracts. Disagreements were resolved by discussion. Two reviewers independently screened full texts. Disagreements were resolved by a third reviewer.

Data were extracted independently by two reviewers using a data collection form. Discrepancies were resolved by discussion and recourse to the original data. A full list of data extracted can be found in the supplementary material (Appendix E).

#### Quality assessment

Two reviewers independently undertook quality assessment. Discrepancies were resolved by discussion. Risk of bias was assessed using the QUADAS (Quality Assessment of Diagnostic Accuracy Studies)-2 tool [27]. We tailored QUADAS-2 to the review by:

- Introducing questions to ensure additional anticipated sources of bias were assessed.
- Altering the ‘Reference Standard’ domain to consider the diagnostic criteria used to identify patients with the bacterial infection being studied.

We developed the tool with a statistician during the protocol stage of the review. It assesses four domains: Patient Selection, Index Test, Reference Standard and Flow and Timing. Each of these domains was scored as low, high or unclear risk of bias (insufficient information to make a judgement) and these data are presented in tabular form. Applicability was not assessed using this tool as only studies meeting review inclusion criteria were considered. An outline of the tool can be found in the supplementary material (Appendix C).

### Analysis

Meta-analysis was undertaken if there were at least three studies reporting the same outcome. Data from studies presenting continuous and dichotomous (e.g. tachycardia) outcomes were analysed separately. We analysed dichotomous outcomes as defined by the studies, although these definitions varied (e.g. >90 or >100 bpm for tachycardia).

Where separate studies reported results from the same cohort of patients, we included only the largest study.

We used meta-regression to assess the relationship between age and vital signs using methodology adapted from Ishak *et al.* [28]. The approach used linear mixed models to account for the correlations, both within and between studies. We included random coefficients in the model to allow for continuous age to be incorporated and for the measurements to be irregularly spaced (according to age). These analyses included all studies, including those only reporting results in one age group. The mean age of each group was used for the purpose of analysis. The mean values for age and associated 95% confidence intervals (95% CI) were plotted along with the individual study values.

Where studies reported vital signs in two or more age groups, we undertook dichotomous meta-analysis in younger and older patients. Where it was necessary to combine subgroups into a single group, we used standard formulae for combining summary statistics across two groups [29]. Groups classified as ‘younger’ patients had a mean age of <65 years or an upper age range of 64 years. Groups classified as ‘older’ patients had a mean age of ≥65 years or a lower age range of 65 years. Sixty five years were selected as this was the most common threshold used in the included studies. Where studies reported median, IQR or range rather than mean and SD, we used methods outlined in the Cochrane Handbook for Systematic Reviews of Interventions to obtain mean and SD [29]. Effect measures were combined using a random effects meta-analysis. The combined results are reported with 95% CI.

Given the broad nature of the question for this systematic review included studies were likely to display clinical heterogeneity (variation in patient location, infections studied, country and age threshold used). Analyses are reported with a measure of between study heterogeneity (the *I*^2^ statistic). Where there was sufficient data, heterogeneity was explored by performing subgroup analysis for different types of bacterial infection (bacteraemia and respiratory tract infections).

Funnel plots were used to assess reporting biases. Statistical analyses were carried out using R software.

## Results

**Figure 1:**
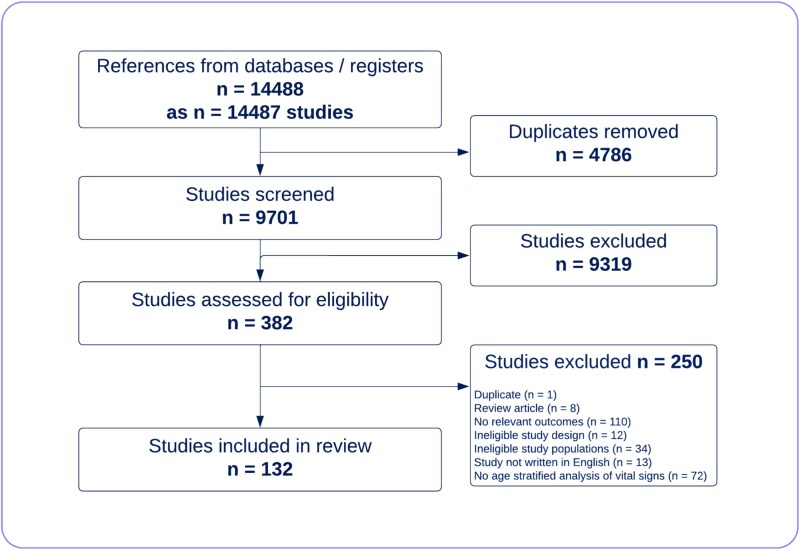
Flow chart showing the process for identification of studies eligible for inclusion.

Of 9701 non-duplicate studies screened, 132 were included (Figure 1). Characteristics of the studies included are shown in Table 1. Most studies were retrospective cohort studies (71 studies, 54%). The studies included 110 475 participants (ranging from 10 to 20 192 per study). Forty three studies (33%) had at least 500 participants. We extracted data from 114 studies of temperature, 61 studies of HR, 43 studies of RR and 49 studies of SBP. Some studies presented data on more than one vital sign and subsequently were included in multiple analyses. Regarding identification of patients with infection, 67 studies used clinical diagnostic criteria (based on history, examination or investigations); 41 studies required microbiologic or pathologic confirmation; 15 studies utilised diagnostic coding; and 9 studies did not state how they identified infection. Characteristics of individual studies can be found in the supplementary material (Appendix D).

**Table 1:** Characteristics of included studies.

Characteristic		Number of studies
**Study Design**	Retrospective cohort	71
	Prospective cohort	37
	Retrospective cross-sectional	13
	Prospective cross-sectional	3
	Retrospective observational	1
	Ambispective observational	1
	Prospective observational	6
**Study continent**	Europe	51
	Asia	38
	North America	37
	Australia	3
	South America	1
	Africa	1
	International	1
**Number of age groups studied**	1	80
	2	32
	3	17
	4	2
	5	1
**Infection studied**	Respiratory	33
	Intra-abdominal	30
	Bacteraemia	21
	Infection	10
	Urinary tract	10
	CNS	9
	Sepsis	10
	Endocarditis	5
	Musculoskeletal	4
**Risk of bias: Domain 1**	High risk of bias	96
Patient selection	Low risk of bias	29
	Unclear risk of bias	7
**Risk of bias: Domain 2**	High risk of bias	58
Index test(s)	Low risk of bias	67
	Unclear risk of bias	7
**Risk of bias: Domain 3**	High risk of bias	6
Reference standard	Low risk of bias	119
	Unclear risk of bias	7
**Risk of bias: Domain 4**	High risk of bias	91
Flow and timing	Low risk of bias	36
	Unclear risk of bias	5

### Risk of bias

Nearly all studies (129/132) were found to be at high risk of bias in at least one domain. Ninety six studies had high risk of bias in domain 1 (patient selection). Fifty eight studies had a high risk of bias in Domain 2 (vital sign measurement). Six studies had a high risk of bias in Domain 3 (definition of target condition). Ninety one studies had high risk of bias in Domain 4 (study flow and timing). Common reasons for being classified as high risk of bias included: failure to pre-specify a threshold for dichotomous/categorical measures, and the retrospective nature of many of the studies. Many studies included small numbers of patients and so were likely to be underpowered to account for measurement variability of vital signs. Risk of bias assessments for individual studies can be found in the supplementary material (Appendix F).

Contour-enhanced funnel plots revealed broadly symmetrical plots. Where asymmetry existed, it was likely due to a combination of poor methodological quality in smaller studies leading to inflated estimates and substantial heterogeneity across all studies (Supplementary Figure S7).

### Heart rate

**Figure 2:**
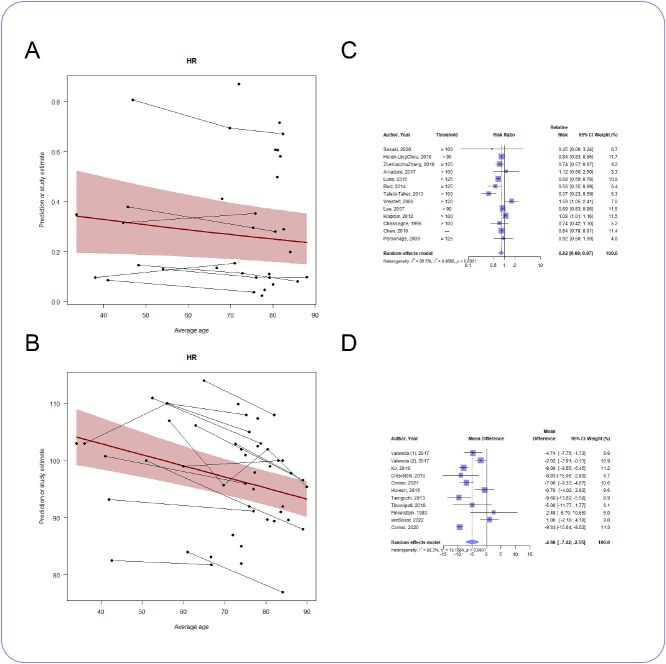
(A) Meta-regression showing how proportion of patients with tachycardia changes with age; *n* = 22 studies (37 762 patients), slope coefficient −0.0095, 95% CI −0.02 to 0.00075, *P* = .069. (B) Meta-regression showing how admission HR changes with age; *n* = 30 studies (22 770 patients), slope coefficient −0.19, 95% CI −0.28 to −0.11, *P* < .05. (C) Forest plot of the risk ratio of tachycardia in older and younger patients; *n* = 13 studies, RR 0.82, 0.69 to 0.97, *I*^2^ = 86.5%. (D) Forest plot of the mean difference in admission HR between older and younger patients; *n* = 11 studies, MD −5 bpm, −7 to −3 bpm, *I*^2^ = 88.3%.

Analysis of admission HR across all ages showed that HR decreased with age (*n* = 30 studies, slope coefficient −0.19, 95% CI −0.28 to −0.11, *P* < .05) (Figure 2b). The change in proportion of patients presenting with tachycardia across all ages was of borderline statistical significance (*n* = 22 studies, slope coefficient −0.0095, 95% CI −0.02 to 0.00075, *P* = .069). (Figure 2a). However, when analysed dichotomously older adults were less likely to present to hospital with tachycardia (*n* = 13 studies, RR 0.82, 95% CI 0.69–0.97, *I*^2^ = 86.5%) (Figure 2c) with a mean difference in HR of −5 bpm (*n* = 11 studies, MD −5 bpm, −7 to −3 bpm, *I*^2^ = 88.3%) (Figure 2d).

### Temperature

**Figure 3:**
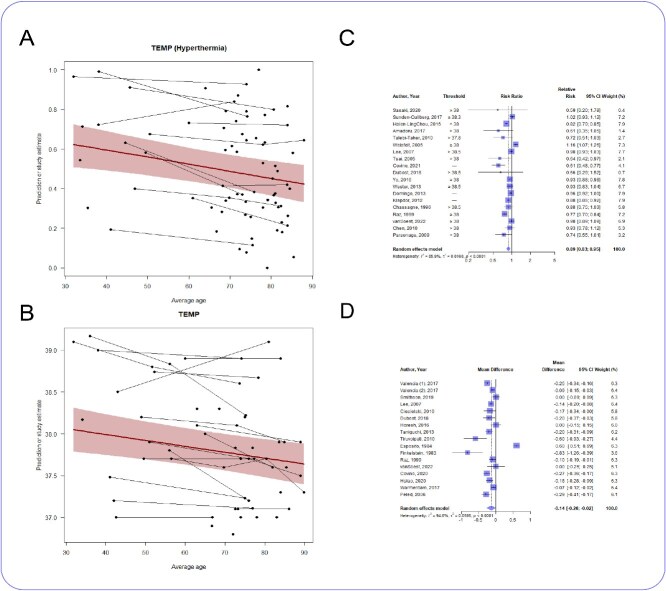
(A) Meta-regression showing how proportion of patients with fever changes with age; *n* = 58 studies (38 696 patients), slope coefficient −0.014, 95% CI −0.024 to −0.005, *P* < .05. (B) Meta-regression showing how admission temperature changes with age; *n* = 31 studies (21 566 patients), slope coefficient −0.0071, 95% CI −0.011 to −0.0027, *P* < .05. (C) Forest plot of the risk ratio of fever in older and younger patients; *n* = 19 studies, RR 0.89, 0.83 to 0.95, *I*^2^ = 85.9%. (D) Forest plot of the mean difference in admission temperature between older and younger patients; *n* = 17 studies, MD −0.14°C, −0.26 to −0.02°C, *I*^2^ = 94.6%.

The proportion of patients presenting with fever decreased with age (*n* = 58 studies, slope coefficient −0.014, 95% CI −0.024 to −0.005, *P* < .05) (Figure 3a). Admission temperature also decreased with age (*n* = 31 studies, slope coefficient − 0.0071, 95% CI −0.011 to −0.0027, *P* < .05) (Figure 3b). Older adults were less likely to present to hospital with a fever (*n* = 19 studies, RR 0.89, 0.83 to 0.95, *I*^2^ = 85.9%) (Figure 3c) with a mean difference in temperature of −0.14°C (*n* = 17 studies, MD −0.14°C, −0.26 to −0.02°C, *I*^2^ = 94.6%) (Figure 3d). There was no relationship between hypothermia and age (*n* = 13 studies, slope coefficient 0.0058 95% CI −0.011 to 0.022, *P* = .49) (Supplementary Figure S2a). Older adults were not more likely to be hypothermic (*n* = 8 studies, RR 1.2, 95% CI 0.91 to 1.57, *I*^2^ = 68.7%) (Supplementary Figure S2b).

### Respiratory rate

**Figure 4:**
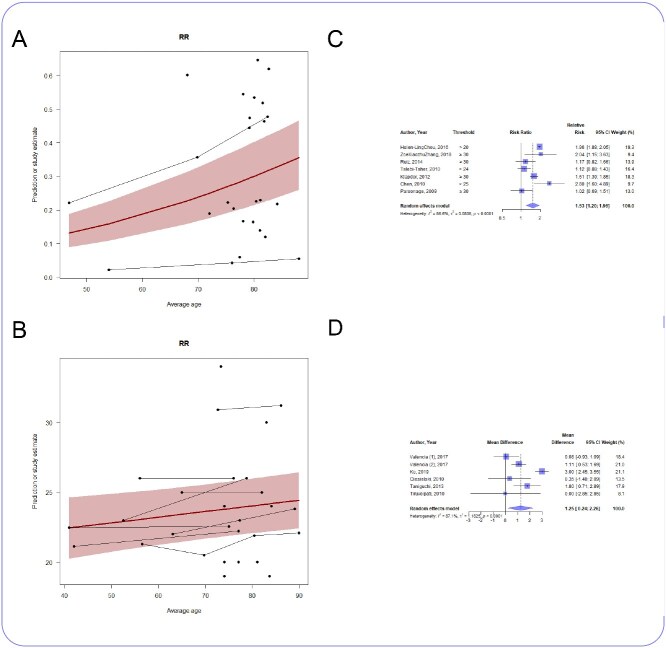
(A) Meta-regression showing how proportion of patients with tachypnoea changes with age; *n* = 20 studies (31 748 patients), slope coefficient 0.032, 95% CI 0.03–0.034, *P* < .05. (B) Meta-regression showing how admission RR changes with age; *n* = 16 studies (11 985 patients), slope coefficient 0.04, 95% CI 0.0084 to 0.072, *P* < .05. (C) Forest plot of the risk ratio of tachypnoea in older and younger patients; *n* = 7 studies, RR 1.53, 1.2 to 1.96, *I*^2^ = 86.6%. (D) Forest plot of the mean difference in admission RR between older and younger patients; *n* = 6 studies, MD 1 bpm, 0–2 bpm, *I*^2^ = 87.1%.

The proportion of patients presenting with tachypnoea increased with age (*n* = 20 studies, slope coefficient 0.032, 95% CI 0.03 to 0.034, *P* < .05) (Figure 4a), as did the admission RR (*n* = 16 studies, slope coefficient 0.04, 95% CI 0.0084 to 0.072, *P* < .05) (Figure 4b). Older adults were more likely to present to hospital with tachypnoea (*n* = 7 studies, RR 1.53, 1.2 to 1.96, *I*^2^ = 86.6%) (Figure 4c) with a mean difference in RR of 1 bpm (*n* = 6 studies, MD 1 bpm, 0 to 2 bpm, *I*^2^ = 87.1%) (Figure 4d).

### Blood pressure

The proportion of patients with hypotension increased with age (*n* = 15 studies, slope coefficient 0.045, 95% CI 0.026 to 0.064, *P* < .05) (Supplementary Figure S1a), but comparison between older and younger age groups did not show a significant difference in occurrence of hypotension (*n* = 7 studies, RR 0.84, 0.49 to 1.44, *I*^2^ = 38.4%) (Supplementary Figure S1c). SBP increased with age (*n* = 26 studies, slope coefficient 0.24, 95% CI 0.16 to 0.32, *P* < .05) (Supplementary Figure S1b) with a mean difference in SBP of 7 mmHg (*n* = 9 studies, MD 7 mmHg, 4 to 9 mmHg, *I*^2^ = 85.3%) (Supplementary Figure S1d). There was no significant relationship between DBP or MAP and patient age (Supplementary Figure S3).

### Oxygen saturation

There was no relationship between admission SaO2 and patient age (*n* = 10 studies, slope coefficient −0.024, 95% CI −0.058 to 0.0096, *P* = .16) (Supplementary Figure S4a). There was no significant difference in admission SaO2 between age groups (*n* = 5 studies, MD −1%, −2 to 0%, *I*^2^ = 95.4%) (Supplementary Figure S4b).

### Subgroup analysis

We conducted a subgroup analysis in patients with bacteraemia to ascertain whether the results of our review held true in a patient population with microbiologically confirmed infection. This analysis was limited by the number of eligible studies. Consistent with our results for all infections, there was a trend towards lower rates of tachycardia in older bacteraemic patients (*n* = 3 studies, RR 0.77, 95% CI 0.57 to 1.03, *I*^2^ = 52.3%) but this was not statistically significant (Supplementary Figure S5a). Older adults with bacteraemia were less likely to have fever (*n* = 4 studies, RR 0.95, 95% CI 0.91 to 0.98, *I*^2^ = 0%) (Supplementary Figure S5b) but were not more likely to be hypothermic (*n* = 3 studies, RR 0.61, 95% CI 0.17 to 2.25, *I*^2^ = 60.1%) (Supplementary Figure S5c).

Given the observed relationship between admission RR and patient age, we conducted a subgroup analysis in patients with respiratory infections. In this subgroup, older adults remained more likely to be tachypnoeic than younger adults (*n* = 5 studies, RR 1.52, 95% CI 1.09 to 2.12, *I*^2^ = 75.1%) (Supplementary Figure S6).

### Heterogeneity

We observed substantial heterogeneity in all vital sign analyses, with most *I*^2^ values exceeding 80%. However, most vital signs showed consistent directional change with age. Differences between study populations (ED vs. Medical Assessment Unit vs. ICU; infections studied; country; age threshold used) likely contributed to this heterogeneity. Subgroup analyses with specific infection types (bacteraemia and respiratory infections) suggested underlying diagnosis as a key source of heterogeneity.

## Discussion

Our results demonstrate that ageing is associated with a different vital sign profile in patients presenting to hospital with bacterial infection. Older adults were less likely to be tachycardic or febrile and more likely to be tachypnoeic. There are several potential explanations for the observed differences.

Firstly, age may be associated with a change in the host response to bacterial infection. The reduced incidence of tachycardia in older patients could reflect a higher incidence of cardiac conduction disease, changes in the response to adrenergic activity or medication for chronic disease. One study demonstrated a decrease in the maximal HR achieved during exercise stress testing in healthy older adults, suggesting ageing is associated with a change in the cardiovascular response to stress [30]. Another study found that trauma patients taking a combination of beta-blocker, calcium channel blocker and Angiotensin-converting enzyme inhibitor/Angiotensin-II receptor blocker had a lower HR compared to patients on no cardiovascular medications, highlighting the role polypharmacy may play in this population [31]. However, it is also likely that our results reflect age-related vital sign differences that are not specific to a response to physiological stress. Older adults have been shown to have a lower resting body temperature, higher RR and higher SBP [32–34]. Therefore, our observation, which ageing is associated with a reduced likelihood of fever, is probably at least in part due to older adults having a lower basal body temperature. However, our results did *not* suggest that older adults admitted with bacterial infection are more likely to be hypothermic than younger adults.

Secondly, our findings may reflect differences in how and when different age groups present to hospital with infection. A mild infection in an older patient may trigger a decompensation (such as a delirium or fall) that requires admission to hospital. In contrast, younger or less frail patients only require admission for severe infections and hence may present with more deranged vital signs. Furthermore, studies have demonstrated that older patients admitted to hospital with non-specific deterioration are more likely to receive a diagnosis of infection without microbiological confirmation [20]. The reduced incidence of tachycardia and fever may therefore be because a smaller proportion of older patients included in our meta-analysis had a true bacterial infection. Nevertheless, in our subgroup analysis of patients with confirmed bacteraemia, older adults remained less likely to have fever and there was a trend towards them being less likely to be tachycardic, supporting a genuine difference in the response to infection in different age groups.

The results of this review should be interpreted with caution. We did not attempt to include unpublished studies so there is a possibility of publication bias. However, this may be less of an issue in observational compared to interventional studies. Almost all the included studies were deemed to be at high risk of bias; reflecting the fact that most studies were retrospective observational studies. There was substantial heterogeneity due to variations in study populations, type of infection and outcomes. Of note, we included studies from over a 40-year period, introducing heterogeneity in diagnostic and therapeutic processes that have changed over this time. The observational nature of the studies means that recording of vital signs was not standardised as it would be in prospective clinical trials. This is important to consider as, e.g. RR is often assessed manually and at risk of inaccuracy [35].

Whilst our review shows evidence of an association between age and presentation vital signs, it does not imply causation between patient age and vital sign differences. Significant differences between older and younger populations (comorbidities, medications, frailty and presentation timing) exist, which may in part explain the findings. We did not include data on frailty which, increasingly in clinical practise, is used rather than age to establish the health status of older adults.

Of note, the mean differences in vital signs between older and younger groups are small (5 bpm for HR, 0.14°C for temperature, 1 bpm for RR), particularly considering the margin of error for these measurements. Whilst statistically significant, it is possible that these differences may be of limited clinical significance.

## Conclusion

Despite these limitations, the consistency of our findings across different analyses, adds weight to the overall conclusion that older adults with infection are less likely to present with tachycardia and fever and more likely to present with tachypnoea. Vital signs form only part of the clinical picture when determining whether infection is likely. However, in the first few hours of admission, before a patient is fully assessed by a clinician, they may be part of the limited available information used to identify patients requiring early escalation and specific investigations. As the delivery of sepsis bundles and higher quality care may be associated with the detection of vital sign abnormalities at presentation, our results suggest that special consideration should be given to the possibility of infection in older patients not presenting with tachycardia or fever.

## Supplementary Material

Supplementary_materials_afaf194
