# Supplementary material for: Is age associated with different vital signs in adults presenting to hospital with bacterial infection? A systematic review and meta-analysis
Source: Age Ageing. 2025 Jul 23;54(7):afaf194. doi: 10.1093/ageing/afaf194 (PMC12284760; doi:10.1093/ageing/afaf194)
Supplement: Supplementary_materials_afaf194 [file supplementary_materials_afaf194.pdf]

Is age associated with different vital signs in adults presenting to hospital with bacterial infection? A systematic review and meta-analysis.

Appendix A: Supplementary Figures
Appendix B: Full Search Strategy
Appendix C: Quality Assessment Tool
Appendix D: Characteristics of Individual Included Studies
Appendix E: Data Collection
Appendix F: Risk of Bias Assessment of Individual Included Studies

Appendix A: Supplementary Figures

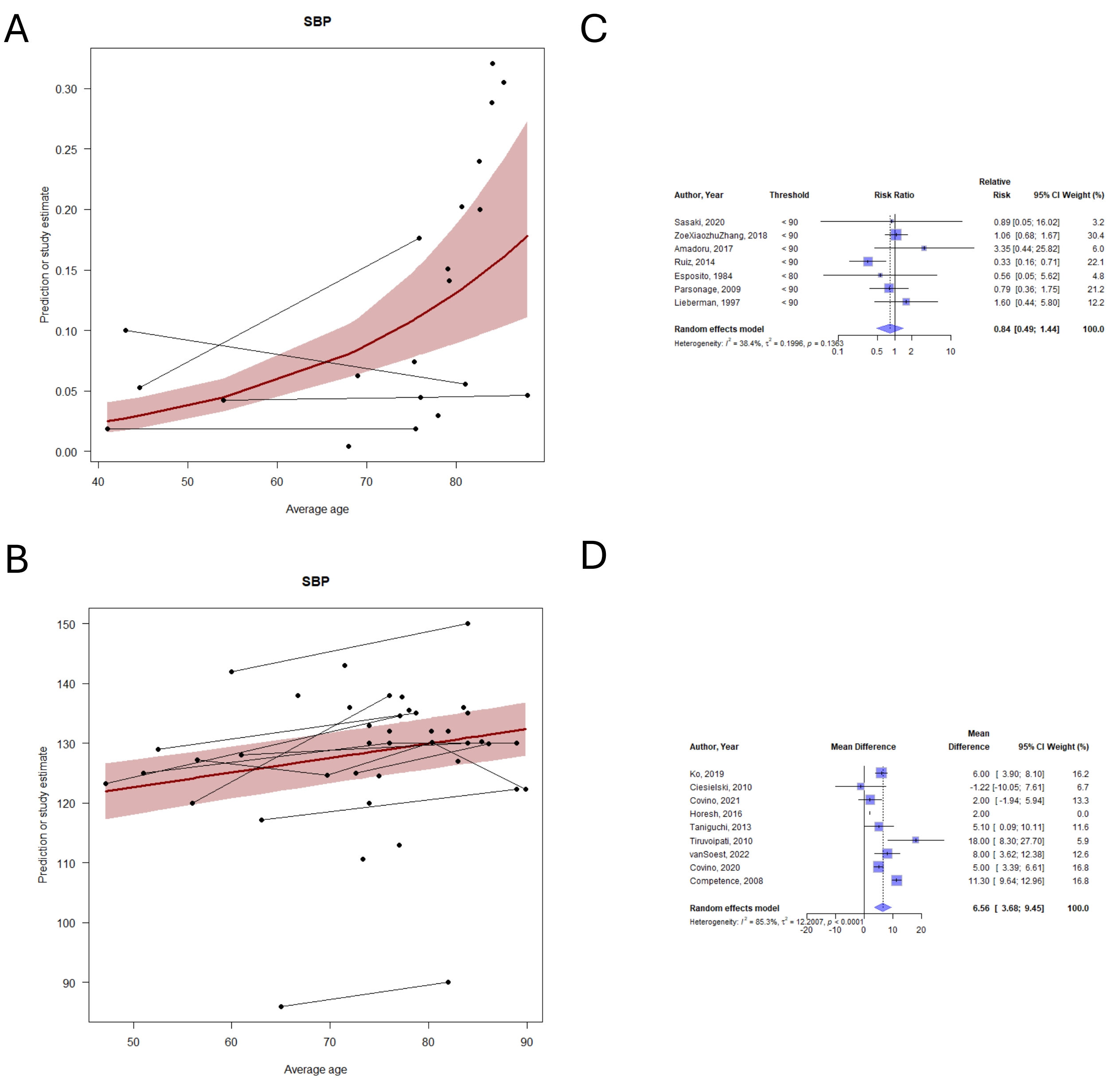

Supplementary Figure 1. [A] Meta-regression showing how proportion of patients with hypotension changes with age; n = 15 studies (6478 patients), slope coefficient 0.045, 95%CI 0.026 to 0.064, p < 0.05. [B] Meta-regression showing how admission systolic blood pressure changes with age; n = 26 studies (21220 patients), slope coefficient 0.24, 95%CI 0.16 to 0.32, p < 0.05. [C] Forest plot of the risk ratio of hypotension in older and younger patients; n = 7 studies, RR 0.84, 0.49 to 1.44, I^2 = 38.4. [D] Forest plot of the mean difference in systolic blood pressure between older and younger patients; n = 9 studies, MD 7mmHg, 4 to 9mmHg, I^2 = 85.3.

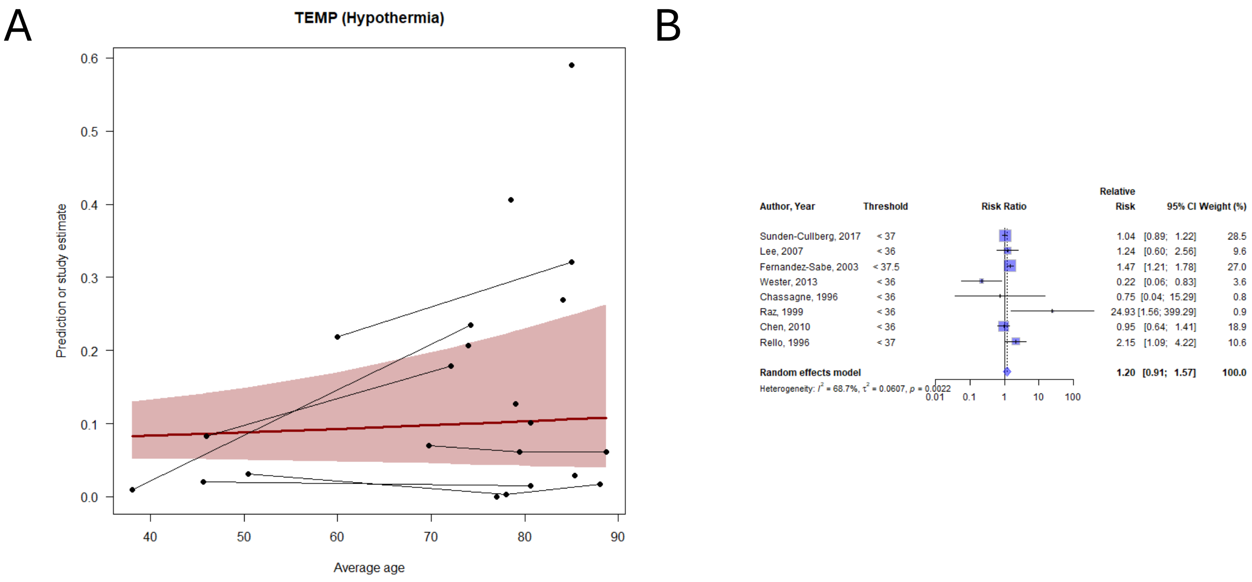

Supplementary Figure 2. [A] Meta-regression showing how proportion of patients with hypothermia changes with age; n = 13 studies (9458 patients), slope coefficient 0.0058 95%CI -0.011 to 0.022, p = 0.49. [B] Forest plot of the risk ratio of hypothermia in older and younger patients; n = 8 studies, RR 1.2, 95%CI 0.91 to 1.57, I^2 = 68.7%).

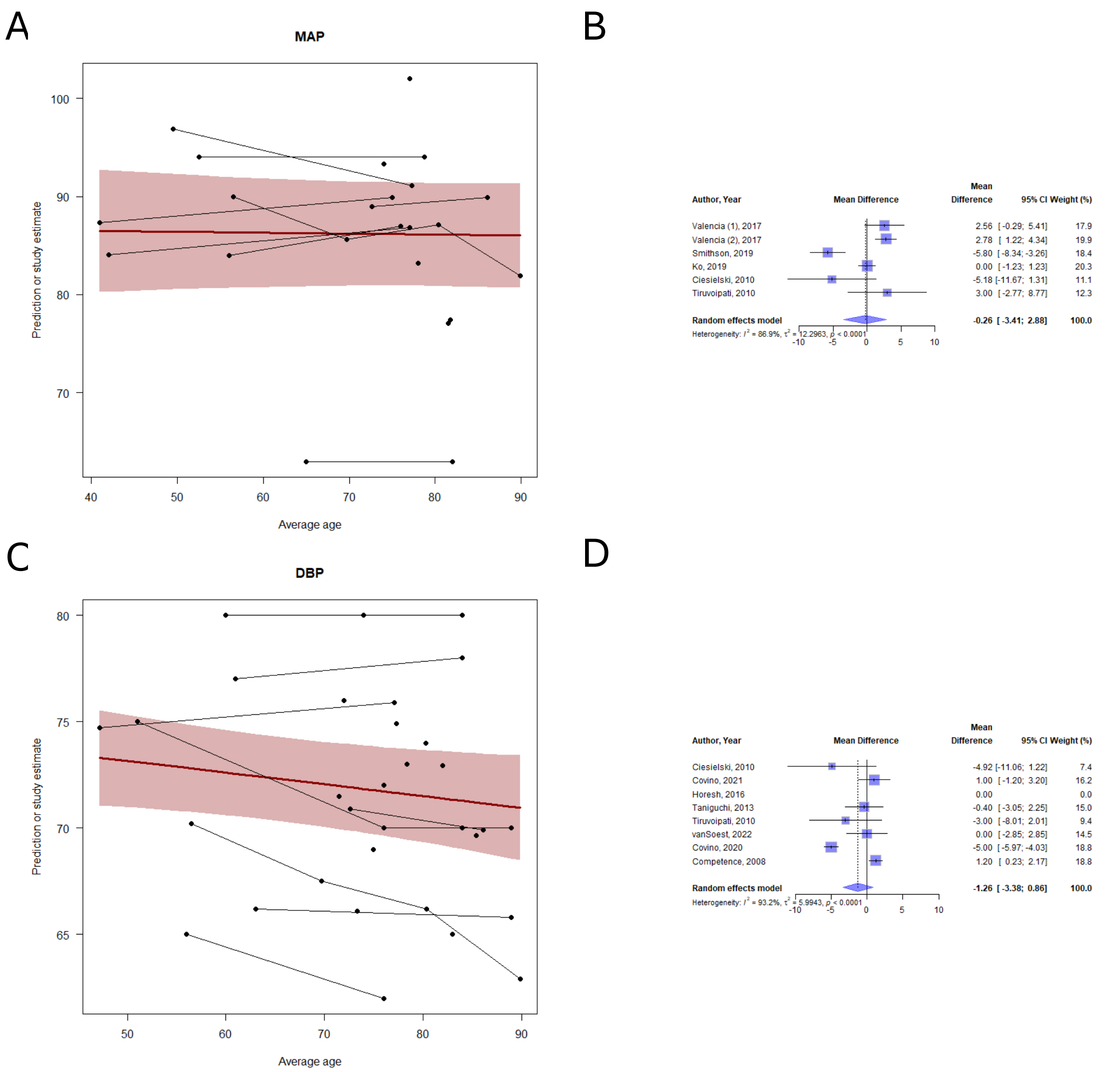

Supplementary Figure 3. [A] Meta-regression showing how admission mean arterial pressure changes with age; n = 11 studies (9797 patients), slope coefficient -0.0095, 95%CI -0.095 to 0.076, p 0.83. [B] Forest plot of the mean difference in mean arterial pressure between older and younger patients; n = 6 studies, MD 0mmHg, -3 to 3mmHg, I^2 = 86.9%. [C] Meta-regression showing how admission diastolic blood pressure changes with age; n = 19 studies (16721 patients), slope coefficient -0.055, 95%CI -0.11 to 0.0039, p = 0.067. [D] Forest plot of the mean difference in diastolic blood pressure between older and younger patients; n = 8 studies, MD -1mmHg, -3 to 1mmHg, I^2 = 93.2%.

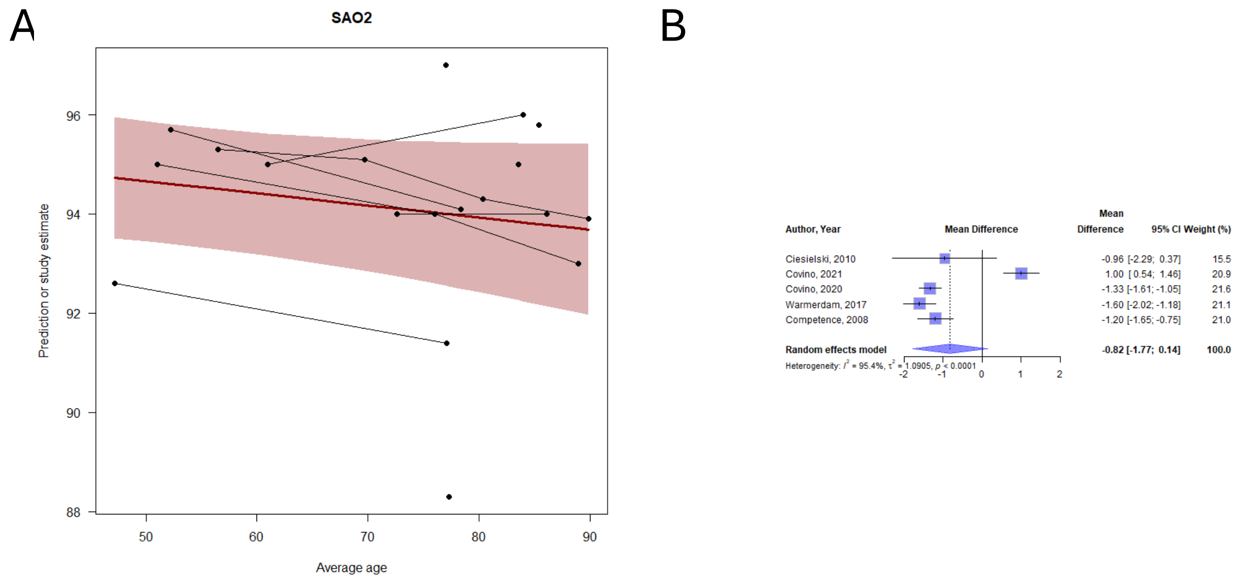

Supplementary Figure 4. [A] Meta-regression showing how admission oxygen saturation changes with age; n = 10 studies (11911 patients), slope coefficient -0.024, 95%CI -0.058 to 0.0096, p = 0.16. [B] Forest plot of the mean difference in oxygen saturation between older and younger patients; n = 5 studies, MD -1%, -2 to 0%, I^2 = 95.4%.

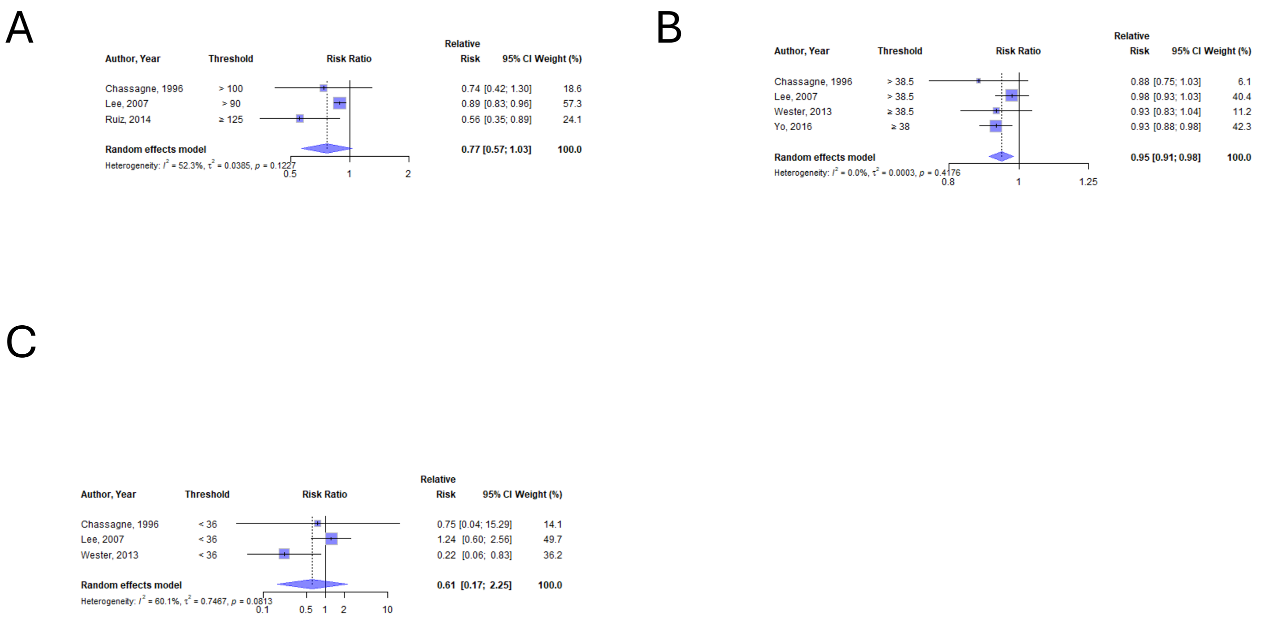

Supplementary Figure 5. [A] Forest plot of the risk ratio of tachycardia in older and younger patients with bacteraemia; n = 3 studies, RR 0.77, 0.57 to 1.03, I^2 = 52.3%. [B] Forest plot of the risk ratio of fever in older and younger patients with bacteraemia; n = 4 studies, RR 0.95, 0.91 to 0.98, I^2 = 0%. [C] Forest plot of the risk ratio of hypothermia in older and younger patients with bacteraemia; n = 3 studies, RR 0.61, 0.17 to 2.25, I^2 = 60.1%.

A

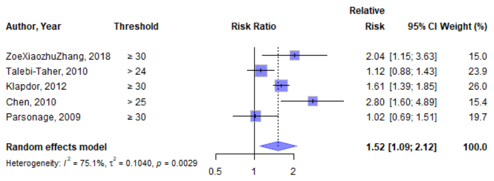

Supplementary Figure 6. [A] Forest plot of the risk ratio of tachypnoea in older and younger patients with respiratory tract infections; n = 5 studies, RR 1.52, 1.09 to 2.12, I^2 = 75.1%.

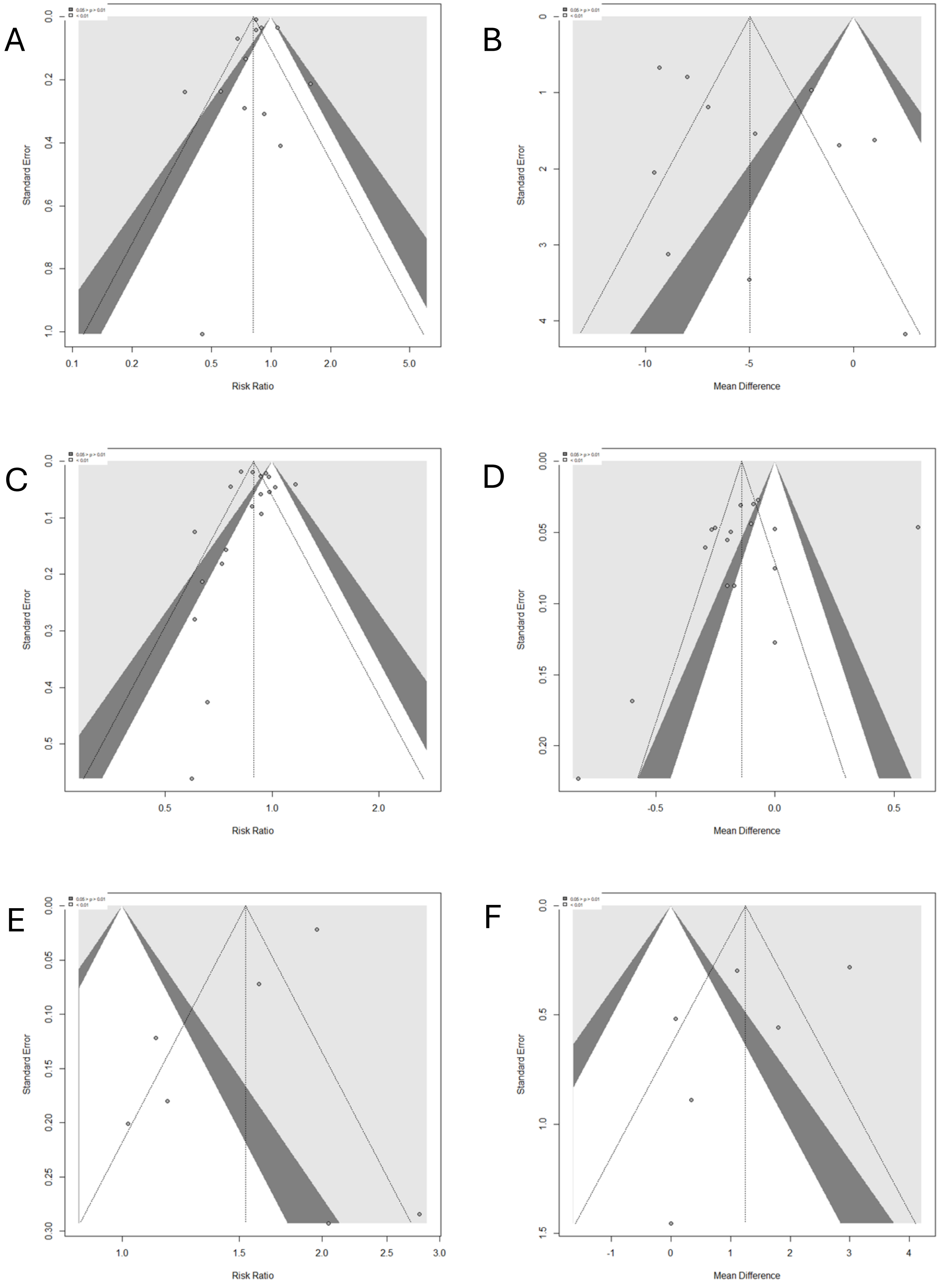

Supplementary Figure 7. Funnel plots for studies reporting [A] HR as dichotomous outcome, [B] HR as continuous outcome, [C] Temp as dichotomous outcome (fever), [D] Temp as continuous outcome, [E] RR as dichotomous outcome, [F] RR as continuous outcome

Appendix B: Full Search Strategy

<u>Search strategy for MEDLINE</u>

Medline (Ovid MEDLINE® Epub Ahead of Print, In-Process & Other Non-Indexed Citations, Ovid MEDLINE® Daily and Ovid MEDLINE®) 1946 to present

#	Search	Results
1	vital signs/ or blood pressure/ or body temperature/ or heart rate/ or respiratory rate/	439176
2	Oximetry/	14418
3	consciousness disorders/ or unconsciousness/	8146
4	Fever/	43208
5	("vital sign*" or "heart rate*" or "respiratory rate*" or "blood pressure*" or "body temperature*" or fever* or febrile or afebrile or "oxygen saturation*" or consciousness or "mental status").ab,ti.	809248
6	bacterial infections/ or bacteremia/ or endocarditis, bacterial/ or gram-negative bacterial infections/ or gram-positive bacterial infections/ or pneumonia, bacterial/ or skin diseases, bacterial/	155733
7	sepsis/ or shock, septic/	91111
8	Urinary Tract Infections/	42646
9	Osteomyelitis/	22078
10	Arthritis, Infectious/	12146
11	intraabdominal infections/ or appendicitis/ or peritonitis/	47196
12	Cholecystitis/	13132
13	Gastroenteritis/	16571
14	Diverticulitis/	3530
15	Cellulitis/	8073
16	Pyelonephritis/	14498
17	Empyema/	4391
18	Fasciitis, Necrotizing/	3304
19	Meningitis, Bacterial/	7749

20	(sepsis or septic*).ab,ti.	176886
21	(bacteremia* or bacteraemia* or septicemia* or septicaemia*).ab,ti.	53968
22	(blood* adj2 infection*).ab,ti.	21237
23	(bacterial adj2 infection*).ab,ti.	57087
24	cellulitis.ab,ti.	10420
25	(((respiratory or chest) adj2 infection*) or (bacterial adj2 pneumonia*)).ab,ti.	62418
26	(((intraabdom* or intra-abdom* or abdom*) adj2 infection*) or appendicitis or diverticulitis or cholecystitis).ab,ti.	49082
27	((urin* adj2 infection*) or pyelonephritis).ab,ti.	63718
28	(bacterial adj2 meningitis).ab,ti.	8219
29	(bacterial adj2 endocarditis).ab,ti.	5248
30	(osteomyelitis or septic arthritis).ab,ti.	30472
31	1 or 2 or 3 or 4 or 5	1040285
32	6 or 7 or 8 or 9 or 10 or 11 or 12 or 13 or 14 or 15 or 16 or 17 or 18 or 19 or 20 or 21 or 22 or 23 or 24 or 25 or 26 or 27 or 28 or 29 or 30	670187
33	31 and 32	51728
34	Age Factors/	472193
35	"Aged, 80 and over"/ or Aged/	3451273
36	Aging/	250314
37	Geriatrics/	31359
38	(aged or geriatric* or elder* or older or ageing or aging or veteran* or septuagenarian* or octogenarian* or nonagenarian* or centenarian*).m_titl	460466
39	34 or 35 or 36 or 37 or 38	4023992
40	33 and 39	11843
41	limit 40 to animals	274
42	40 not 41	11569
43	"case report* ".m_titl.	326807
44	Case Reports/	2340387
45	43 or 44	2393510
46	42 not 45	9011

47	child/ or infant/ or infant, newborn/	2643647
48	Fetus/	82959
49	Child, Preschool/	988951
50	(paediatric* or pediatric* or child* or boy* or girl* or infant* or neonat* or newborn*).m_titl.	1482670
51	47 or 48 or 49 or 50	3136310
52	46 not 51	7187
53	limit 52 to english language	6205

Search strategy for EMBASE

1974 to present

#	Search	Results
1	*vital sign/	1923
2	*blood pressure/	81247
3	*body temperature/	11758
4	*heart rate/	49545
5	*breathing rate/	4355
6	*pulse oximetry/ or *oximetry/	6609
7	*consciousness/ or *"altered state of consciousness"/ or *consciousness level/ or *consciousness disorder/	10850
8	*unconsciousness/	2160
9	("vital sign*" or "heart rate*" or "respiratory rate*" or "blood pressure*" or "body temperature*" or fever* or febrile or afebrile or "oxygen saturation*" or consciousness or "mental status").ab,ti.	1165800
10	1 or 2 or 3 or 4 or 5 or 6 or 7 or 8 or 9	1215846
11	*bacterial infection/	61934

12	*bacteremia/	17294
13	*bacterial endocarditis/	19830
14	*bacterial pneumonia/	6068
15	*sepsis/	61539
16	*septic shock/	22111
17	*urinary tract infection/	40002
18	*osteomyelitis/	14937
19	*bacterial arthritis/	5133
20	*abdominal infection/	2267
21	*appendicitis/ or *acute appendicitis/	16456
22	*bacterial peritonitis/	3473
23	*cholecystitis/ or *acute cholecystitis/	10868
24	*acute gastroenteritis/ or *gastroenteritis/	11179
25	*diverticulitis/	3742
26	*cellulitis/	4929
27	*pyelonephritis/ or *acute pyelonephritis/	8878
28	*empyema/	3772
29	*necrotizing fasciitis/	3914
30	*bacterial meningitis/	10923
31	(sepsis or septic*).ab,ti.	264600
32	(bacteremia* or bacteraemia* or septicemia* or septicaemia*).ab,ti.	70881
33	(blood* adj2 infection*).ab,ti.	32211
34	(bacterial adj2 infection*).ab,ti.	78456
35	cellulitis.ab,ti.	15612
36	(((respiratory or chest) adj2 infection*) or (bacterial adj2 pneumonia*)).ab,ti.	94907
37	(((intraabdom* or intra-abdom* or abdom*) adj2 infection*) or appendicitis or diverticulitis or cholecystitis).ab,ti.	62022

38	((urin* adj2 infection*) or pyelonephritis).ab,ti.	93343
39	(bacterial adj2 meningitis).ab,ti.	10333
40	(bacterial adj2 endocarditis).ab,ti.	4674
41	(osteomyelitis or septic arthritis).ab,ti.	36318
42	11 or 12 or 13 or 14 or 15 or 16 or 17 or 18 or 19 or 20 or 21 or 22 or 23 or 24 or 25 or 26 or 27 or 28 or 29 or 30 or 31 or 32 or 33 or 34 or 35 or 36 or 37 or 38 or 39 or 40 or 41	771402
43	10 and 42	76772
44	age/	587564
45	aged/	3589886
46	aging/	314680
47	geriatrics/	33957
48	(aged or geriatric* or elder* or older or ageing or aging or veteran* or septuagenarian* or octogenarian* or nonagenarian* or centenarian*).m_titl.	581574
49	44 or 45 or 46 or 47 or 48	4320400
50	43 and 49	14585
51	limit 50 to animals	58
52	50 not 51	14527
53	"case report* ".m_titl.	404093
54	case report/	2913725
55	53 or 54	2942608
56	52 not 55	9569
57	child/	2105718
58	infant/	697896
59	fetus/	214751
60	(paediatric* or pediatric* or child* or boy* or girl* or infant* or neonat* or newborn*).m_titl.	1766639
61	57 or 58 or 59 or 60	3224298
62	56 not 61	7954
63	limit 62 to english language	7199
64	limit 63 to conference abstract	1623
65	63 not 64	5576

Search strategy for CINAHL

#	Search	Results
1	MH vital signs	3263
2	MH blood pressure	46211
3	MH body temperature	4721
4	MH heart rate	33243
5	MH respiratory rate	2414
6	MH pulse oximetry	2438
7	MH consciousness	3739
8	MH consciousness disorders	1119
9	TI ("vital sign*" or "heart rate*" or "respiratory rate*" or "blood pressure*" or "body temperature*" or fever* or febrile or afebrile or "oxygen saturation*" or consciousness or "mental status") OR AB ("vital sign*" or "heart rate*" or "respiratory rate*" or "blood pressure*" or "body temperature*" or fever* or febrile or afebrile or "oxygen saturation*" or consciousness or "mental status")	182239
10	S1 OR S2 OR S3 OR S4 OR S5 OR S6 OR S7 OR S8 OR S9	217578
11	MH bacterial infection	0
12	MH bacterial infection	845
13	MH bacteremia	6405
14	MH endocarditis	2314
15	MH bacterial pneumonia	0
16	MH pneumonia	12585
17	MH sepsis or septic or severe sepsis or septic shock	31198
18	MH urinary tract infection	0

19	MH pyelonephritis	1216
20	MH osteomyelitis	4282
21	MH septic arthritis	0
22	MH appendicitis	3984
23	MH cholecystitis	1658
24	MH diverticulitis	867
25	MH gastroenteritis	3074
26	MH cellulitis	1943
27	MH empyema	908
28	MH necrotizing fasciitis	0
29	MH meningitis	3188
30	TI (sepsis or septic*) OR AB (sepsis or septic*)	38865
31	TI ("urinary tract infection*") OR AB ("urinary tract infection*")	11404
32	TI (bacteremia* or bacteraemia* or septicemia* or septicaemia*) OR AB (bacteremia* or bacteraemia* or septicemia* or septicaemia*)	8314
33	TI (meningitis) OR AB (meningitis)	8357
34	TI (osteomyelitis) or AB (osteomyelitis)	4941
35	TI ("infectious arthritis" or "septic arthritis") OR AB ("infectious arthritis" or "septic arthritis")	1600
36	TI (endocarditis) OR AB (endocarditis)	6557
37	TI (appendicitis OR cholecystitis OR diverticulitis) OR AB (appendicitis OR cholecystitis OR diverticulitis)	7627
38	TI (cellulitis) OR AB (cellulitis)	2552
39	TI (pneumonia) OR AB (pneumonia)	34001
40	TI (pyelonephritis) OR AB (pyelonephritis)	1497
41	S11 OR S12 OR S13 OR S14 OR S15 OR S16 OR S17 OR S18 OR S19 OR S20 OR S21 OR S22 OR S23 OR S24 OR S25 OR S26 OR S27 OR S28 OR S29 OR S30 OR S31 OR S32 OR S33 OR S34 OR S35 OR S36 OR S37 OR S38 OR S39 OR S40	131265
42	S10 AND S41	11722
43	MH aged	912862

44	MH aging	54759
45	MH geriatric	0
46	TI (aged or geriatric* or elder* or older or ageing or aging or veteran* or septuagenarian* or octogenarian* or nonagenarian* or centenarian*)	233636
47	S43 OR S44 OR S45 OR S46	1017207
48	S42 AND S47	2536
49	TI ((paediatric* or pediatric* or child* or boy* or girl* or infant* or neonat* or newborn*)	535064
50	S48 NOT S49	2476
51	TI ("case report* ")	71100
52	S50 NOT S51	2324
53	S50 NOT S51, limiters – English Language	2292

Appendix C: Quality Assessment Tool

Risk of bias was assessed using an adapted version of the QUADAS (Quality Assessment of Diagnostic Accuracy Studies)-2 tool. It assesses four domains: Patient Selection, Index Test, Reference Standard and Flow and Timing. Each of these domains was scored separately as low risk of bias, high risk of bias or unclear risk of bias (insufficient information to make a judgement).

Domain 1: Patient Selection

1. Was a consecutive or random sample of patients enrolled?

Studies should describe their method of patient enrolment. If a random sample of patients was used, the method of randomisation should be stated. Convenience sampling or arbitrary recruitment introduces a high risk of bias.

2. Did the study avoid inappropriate exclusions?

Exclusion criteria should be clearly justified.

3. Was the sample size justified?

This prompt only applies to prospective studies

4. Are the characteristics of the study population presented in a table or clearly described?

Could the selection of patients have introduced bias?

Low risk of bias: Q1, Q2, Q3, Q4 all answered "yes"
High risk of bias: Any of Q1, Q2, Q3, Q4 answered "no"
Unclear risk of bias: insufficient data reported to permit a judgement

Domain 2: Index test(s)

1. Were the measurements taken by a healthcare professional or trained personnel?

All studies included in the systematic review are in hospitalised patients; therefore, assume vital signs taken by a healthcare professional.

2. Is a description of the measurement technique included?

The study should either describe sufficient and unambiguous details of the measurement techniques used for vital sign recording; or should state where the measurement was obtained (eg. Patient notes).

3. If a threshold was used, was it pre-specified?

Thresholds for study definitions of tachycardia, bradycardia, hypertension, hypotension, tachypnoea, hypoxia, pyrexia and reduced level of consciousness should be pre-specified. Not relevant for continuous outcomes.

Could the conduct or interpretation of the index test have introduced bias?

Low risk of bias: Q1, Q2, Q3 all answered "yes"
High risk of bias: Any of Q1, Q2, Q3 answered "no"
Unclear risk of bias: insufficient data reported to permit a judgement

Domain 3: Reference standard

1. Are the criteria used likely to correctly classify the target condition?

Could the reference standard, its conduct, or its interpretation have introduced bias?

Low risk of bias: Q1 answered "yes"

High risk of bias: Q1 answered “no”
Unclear risk of bias: insufficient data reported to permit a judgement

Domain 4: Flow and Timing

1. Was the study a prospective study?
2. Was there an appropriate interval between vital sign(s) being taken and diagnosis of bacterial infection?
3. Were all patients included in the analysis?

Could the patient flow have introduced bias?

Low risk of bias: Q1, Q2, Q3 all answered “yes”
High risk of bias: Any of Q1, Q2, Q3 answered “no”
Unclear risk of bias: insufficient data reported to permit a judgement

-

Appendix D: Characteristics of Individual Included Studies

Author	Year	Citation	Country	Design	Infection	Infection Definition	Participants	% Female	Age				
									mean	median	sd	lower	upper
Alpay	2018	[1]	Turkey	Retrospective cohort	UTI	Positive microbiology or pathology	140	53.6	78.50	-	-	65	98
Amadoru	2017	[2]	Australia	Retrospective cohort	Spinal	Clinical diagnosis	53	32.1	64.70	-	-	19	92
Applefeld	1974	[3]	United States	Retrospective cohort	Endocarditis	Clinical diagnosis	29	-	-	-	-	-	-
Artero (1)	2016	[4]	Spain	Retrospective observational	UTI	Positive microbiology or pathology	196	50.5	81.74	-	6.62	-	-
Artero (2)	2016	[4]	Spain	Retrospective observational	Bacteraemia	Positive microbiology or pathology	137	62	81.52	-	7.07	-	-
Barsic	1992	[5]	Croatia	Retrospective cohort	Meningitis	Positive microbiology or pathology	70	27.1	-	-	-	8	88
Boonmee	2020	[6]	Thailand	Retrospective cohort	Sepsis	Clinical diagnosis	1180	52.9	-	-	-	-	-
Burns	1991	[7]	United Kingdom	Retrospective cohort	Endocarditis	Clinical diagnostic coding	19	42.1	74.00	-	-	65	84
Cabellos	2009	[8]	Spain	Prospective cohort	Meningitis	Clinical diagnosis	185	58.9	73.00	-	6.00	-	-
Cataudella	2017	[9]	Italy	Prospective cohort	CAP	Clinical diagnosis	195	38.5	80.30	-	7.50	-	-
Caterino	2019	[10]	United States	Prospective observational	Infection	Clinical diagnosis	77	57	74.00	-	7.60	-	-
Caterino (1)	2009	[11]	United States	Prospective cohort	Infection	Clinical diagnosis	935	57	79.10	-	8.30	-	-
Caterino (2)	2009	[11]	United States	Prospective cohort	Infection	Clinical diagnosis	2016	55.6	79.20	-	8.50	-	-
Caterino	2012	[12]	United States	Prospective cohort	Infection	Clinical diagnosis	105	53.3	-	-	-	-	-
Celikhisar	2020	[13]	Turkey	Retrospective cohort	CAP	Clinical diagnosis	86	72.1	73.30	-	19.30	-	-
Chassagne	1996	[14]	France	Prospective cohort	Bacteraemia	Positive microbiology or pathology	105	49.5	69.30	-	-	17	96
Ciesielski	2010	[15]	United States	Retrospective cohort	UTI	Positive microbiology or pathology	270	64	76.16	-	12.80	-	-
Covino	2021	[16]	Italy	Retrospective cohort	Diverticulitis	Clinical diagnostic coding	1139	52	-	67.0	-	-	-
Domingo	2013	[17]	Spain	Prospective observational	Meningitis	Positive microbiology or pathology	635	52.1	-	-	-	-	-
Dubost	2018	[18]	France	Retrospective cohort	Spinal	Clinical diagnosis	152	33	-	-	-	-	-
Elangovan	1996	[19]	United States	Retrospective cross-sectional	Appendicitis	Positive microbiology or pathology	74	40	68.90	-	-	-	-
Erdem	2010	[20]	Turkey	Retrospective cohort	Meningitis	Clinical diagnosis	159	43.4	63.00	-	9.00	-	-
Esposito	1984	[21]	United States	Prospective cohort	Bacteraemia	Positive microbiology or pathology	38	60.5	61.00	-	-	-	-
Ewig (1)	2012	[22]	Germany	Prospective cohort	NHAP	Clinical diagnosis	518	57.3	-	83.3	-	-	-

									Age				
Author	Year	Citation	Country	Design	Infection	Infection Definition	Participants	% Female	mean	median	sd	lower	upper
Ewig (2)	2012	[22]	Germany	Prospective cohort	CAP	Clinical diagnosis	2569	37.4	-	76.1	-	-	-
Ewig	1999	[23]	Germany	Prospective cohort	CAP	Clinical diagnosis	168	58.9	78.00	-	8.00	-	-
Fernandez-Sabe	2003	[24]	Spain	Prospective cohort	CAP	Clinical diagnosis	1474	30.1	-	-	-	-	-
Finkelstein	1983	[25]	United States	Retrospective cohort	Bacteraemia	Positive microbiology or pathology	187	-	-	-	-	20	93
Fontanarosa	1992	[26]	United States	Retrospective cross-sectional	Bacteraemia	Positive microbiology or pathology	79	57	80.60	-	-	68	94
Garcia-Ordonez	2001	[27]	Spain	Retrospective cohort	CAP	Clinical diagnosis	343	42	76.30	-	7.30	-	-
Golcuk	2015	[28]	Turkey	Prospective cross-sectional	CAP	Clinical diagnosis	100	40	77.30	-	7.60	-	-
Gopal	2015	[29]	India	Retrospective cohort	UTI	Clinical diagnosis	100	47	71.50	-	16.30	-	-
Guirgis	2018	[30]	United States	Prospective observational	Sepsis	Clinical diagnosis	10	50	77.00	-	2.00	-	-
Hernandez	2015	[31]	Spain	Prospective cohort	Bacteraemia	Positive microbiology or pathology	2605	47.1	78.06	-	7.71	-	-
Horattas	1990	[32]	United States	Retrospective cohort	Appendicitis	Positive microbiology or pathology	96	49	-	-	-	-	-
Horesh	2016	[33]	Israel	Retrospective cross-sectional	Diverticulitis	Not stated	636	56.4	-	60.0	-	20	98
Hsien-LingChou	2016	[34]	Taiwan	Retrospective cohort	Bacteraemia	Clinical diagnostic coding	20192	45.7	62.00	-	18.60	-	-
Jung	2017	[35]	South Korea	Retrospective cohort	Appendicitis	Clinical diagnostic coding	103	49.5	-	72.0	-	65	89
Kaser	2013	[36]	Switzerland	Retrospective cohort	Diverticulitis	Clinical diagnosis	282	47.5	66.70	-	10.80	-	-
Kijsirichareanchai	2015	[37]	United States	Retrospective cohort	Diverticulitis	Clinical diagnostic coding	94	51.1	45.50	-	15.30	-	-
Kim	2013	[38]	South Korea	Retrospective cohort	NHAP	Clinical diagnosis	73	53.4	79.80	-	9.03	-	-
Kim	2015	[39]	South Korea	Retrospective cohort	Sepsis	Clinical diagnostic coding	458	47.2	-	78.0	-	-	-
Klapdor	2012	[40]	Europe	Prospective cohort	CAP	Clinical diagnosis	7803	44.4	60.90	-	18.50	-	-
Ko	2019	[41]	Netherlands	Prospective cohort	Infection	Clinical diagnosis	2659	43	62.10	-	17.10	-	-
Laborde	2021	[42]	France	Retrospective cohort	Bacteraemia	Positive microbiology or pathology	105	61.9	85.30	-	5.90	-	-
Lee	2007	[43]	Taiwan	Prospective cohort	Bacteraemia	Positive microbiology or pathology	890	48.5	-	-	-	-	-
Lee	2013	[44]	South Korea	Prospective cohort	Sepsis	Clinical diagnosis	401	50.1	-	74.0	-	-	-
Lee	2020	[45]	South Korea	Retrospective cohort	Cholecystitis	Clinical diagnosis	303	46.9	-	74.0	-	-	-

Author	Year	Citation	Country	Design	Infection	Infection Definition	Participants	% Female	Age mean	Age median	Age sd	Age lower	Age upper
Lee	2020	[46]	South Korea	Retrospective cohort	Bacteraemia	Positive microbiology or pathology	186	90.3	-	75.0	-	-	-
Liu	2010	[47]	Australia	Prospective cohort	Infection	Clinical diagnosis	34	62.5	85.40	-	-	71	98
Luna	2016	[48]	International	Retrospective cohort	CAP	Clinical diagnosis	6205	38.8	66.50	-	17.90	-	-
Ma	2011	[49]	China	Prospective cohort	CAP	Clinical diagnosis	488	42.2	81.00	-	7.90	-	-
Marinella	2000	[50]	United States	Retrospective cohort	Diverticulitis	Clinical diagnosis	21	19	34.10	-	5.90	-	-
Meyers	1989	[51]	United States	Retrospective cohort	Bacteraemia	Positive microbiology or pathology	100	63	-	-	-	-	-
Myint	2005	[52]	United Kingdom	Prospective cohort	CAP	Clinical diagnosis	100	44	-	81.5	-	65	96
Mylotte	2002	[53]	United States	Retrospective cohort	Bacteraemia	Positive microbiology or pathology	169	64.5	82.70	-	8.10	-	-
Parker	1997	[54]	United States	Retrospective cross-sectional	Cholecystitis	Clinical diagnosis	168	48.2	74.00	-	-	65	93
Potts	1999	[55]	United States	Retrospective cross-sectional	Intra-abdominal	Clinical diagnosis	117	52.1	85.50	-	-	80	97
Rasmussen	1992	[56]	Denmark	Retrospective cohort	Meningitis	Clinical diagnosis	48	64.6	-	69.0	-	-	-
Raz	1999	[57]	Israel	Retrospective cohort	UTI	Positive microbiology or pathology	201	100	61.00	-	22.10	-	-
Riquelme	1996	[58][59]	Spain	Prospective cohort	CAP	Clinical diagnosis	101	33.7	78.00	-	8.00	-	-
Robbins	1980	[60]	United States	Retrospective cohort	Endocarditis	Clinical diagnostic coding	56	36	71.70	-	-	65	92
Ruiz	2014	[61]	Spain	Prospective cohort	Bacteraemia	Positive microbiology or pathology	399	32.8	-	-	-	-	-
Ryden	1983	[62]	Sweden	Retrospective cohort	Appendicitis	Clinical diagnosis	672	-	-	-	-	-	-
Salahuddin	2012	[63]	Pakistan	Prospective observational	Appendicitis	Positive microbiology or pathology	36	44	65.50	-	4.20	-	-
Sasaki	2020	[64]	Japan	Retrospective cohort	Diverticulitis	Clinical diagnosis	237	34.2	44.80	-	-	-	-
Sheu	2007	[65]	Taiwan	Retrospective cohort	Appendicitis	Clinical diagnostic coding	601	52	69.90	-	7.50	-	-
Shimazui	2020	[66]	Japan	Retrospective cohort	Sepsis	Clinical diagnosis	1148	39.6	-	-	-	-	-
Shimoni	2021	[67]	Israel	Retrospective cross-sectional	Bacteraemia	Positive microbiology or pathology	222	58.6	84.00	-	8.00	-	-
Singler	2013	[68]	Germany	Prospective observational	Infection	Clinical diagnosis	105	64.8	83.60	-	4.70	-	-
Sirikurnpiboon	2015	[69]	Thailand	Retrospective cohort	Appendicitis	Positive microbiology or pathology	206	62.1	68.98	-	7.08	-	-

Author	Year	Citation	Country	Design	Infection	Infection Definition	Participants	% Female	Age				
									mean	median	sd	lower	upper
Smithson	2019	[70]	Spain	Ambispective observational	UTI	Positive microbiology or pathology	552	0	66.10	-	16.70	-	-
Sunden-Cullberg	2017	[71]	Sweden	Prospective cohort	Sepsis	Clinical diagnostic coding	2225	44	-	68.0	-	-	-
Talebi-Taher	2010	[72]	Iran	Prospective cohort	CAP	Clinical diagnosis	183	-	59.00	-	24.00	-	-
Taniguchi	2013	[73]	Japan	Retrospective cross-sectional	Bacteraemia	Positive microbiology or pathology	366	63.4	73.20	-	20.30	-	-
Tantarattanapong	2018	[74]	Thailand	Retrospective cohort	Appendicitis	Positive microbiology or pathology	223	55.6	-	68.0	-	-	-
Tantarattanapng	2021	[75]	Thailand	Retrospective cross-sectional	Sepsis	Clinical diagnosis	600	50.8	-	78.0	-	-	-
Terpenning	1987	[76]	United States	Retrospective cohort	Endocarditis	Positive microbiology or pathology	154	31.2	-	-	-	-	-
Thiem	2009	[77]	Germany	Retrospective cohort	CAP	Clinical diagnostic coding	391	51.2	80.00	-	8.00	-	-
Tiruvoipati	2010	[78]	Australia	Retrospective cohort	Sepsis	Clinical diagnosis	175	46.9	-	-	-	-	-
Tsai	2005	[79]	Taiwan	Retrospective cohort	Empyema	Clinical diagnosis	132	17	-	58.5	-	18	89
Valencia (1)	2017	[80]	Colombia	Prospective cohort	Sepsis	Clinical diagnostic coding	765	50.7	51.62	-	19.96	-	-
Valencia (2)	2017	[80]	Colombia	Prospective cohort	Sepsis	Clinical diagnostic coding	1846	52.3	55.86	-	20.90	-	-
Vincent	1990	[81]	Canada	Retrospective cohort	Septic arthritis	Clinical diagnosis	21	47.6	72.30	-	-	60	92
Warmerdam	2018	[82]	Netherlands	Prospective cohort	Infection	Clinical diagnosis	833	37.6	78.40	-	6.30	-	-
Wasserman	1989	[83]	United States	Prospective cross-sectional	Infection	Positive microbiology or pathology	33	68	82.00	-	-	-	-
Weisfelt	2006	[84]	Netherlands	Prospective cohort	Meningitis	Positive microbiology or pathology	696	50.4	50.20	-	19.96	-	-
Wester	2013	[85]	Norway	Retrospective cohort	Bacteraemia	Positive microbiology or pathology	680	57.5	-	75.0	-	-	-
Yahav	2015	[86]	Israel	Prospective cross-sectional	Bacteraemia	Clinical diagnosis	4308	-	-	-	-	-	-
Yo	2016	[87]	Taiwan	Retrospective cohort	Bacteraemia	Positive microbiology or pathology	937	47.9	63.00	-	17.44	-	-
ZoeXiaozhuZhang	2018	[88]	Singapore	Retrospective cohort	CAP	Clinical diagnosis	1902	44.5	-	73.0	-	-	-
Andersson	1978	[89]	Sweden	Retrospective cohort	Appendicitis	Not stated	68	48.5	68.54	-	-	60	83
Barkham	1996	[90]	United Kingdom	Retrospective cross-sectional	Bacteraemia	Positive microbiology or pathology	104	71.2	-	-	-	-	-
CAP-ChinaNetwork	2020	[91]	China	Retrospective cohort	CAP	Not stated	3011	45.5	77.39	-	7.41	-	-

Author	Year	Citation	Country	Design	Infection	Infection Definition	Participants	% Female	Age				
									mean	median	sd	lower	upper
Chen	2010	[92]	Taiwan	Prospective observational	CAP	Clinical diagnosis	987	38.1	68.00	-	19.30	-	-
Chodak	1981	[93]	United States	Retrospective cohort	Diverticulitis	Clinical diagnosis	37	10.8	-	-	-	21	40
Choi	2022	[94]	United States	Prospective cohort	UTI	Clinical diagnosis	61	59	-	81.1	-	-	-
Cilloniz (1)	2023	[95]	Spain	Prospective cohort	CAP	Clinical diagnosis	1802	42	-	84.0	-	-	-
Cilloniz (2)	2023	[95]	Spain	Prospective cohort	CAP	Clinical diagnosis	204	32	-	83.0	-	-	-
Conte	1999	[96]	United States	Retrospective cohort	CAP	Clinical diagnostic coding	2356	51.5	-	-	-	-	-
Cooper	1986	[97]	United Kingdom	Retrospective cohort	Septic arthritis	Clinical diagnosis	21	42.9	73.80	-	7.00	-	-
Cooper	1994	[98]	United States	Retrospective cohort	Intra-abdominal	Positive microbiology or pathology	131	-	-	-	-	-	-
Covino	2020	[99]	Italy	Retrospective cohort	CAP	Clinical diagnosis	4056	45	-	76.0	-	-	-
Gorse	1984	[100]	United States	Retrospective cohort	Meningitis	Positive microbiology or pathology	86	-	-	-	-	-	-
Habibi	2021	[101]	Iran	Retrospective cross-sectional	CAP	Clinical diagnosis	221	28.5	81.00	-	6.00	-	-
deGroot	2017	[102]	Netherlands	Retrospective cohort	Infection	Clinical diagnosis	2280	42.3	61.10	-	17.00	-	-
Hall	1976	[103]	United States	Retrospective cross-sectional	Appendicitis	Not stated	50	46	-	-	-	60	95
Hsiao	2020	[104]	Taiwan	Retrospective cohort	UTI	Positive microbiology or pathology	1043	72.9	67.00	-	17.00	-	-
Hui	2002	[105]	United States	Retrospective cohort	Appendicitis	Not stated	95	51.6	78.00	-	5.60	-	-
Joo	2022	[106]	Canada	Retrospective cohort	UTI	Clinical diagnosis	129	58.1	85.50	-	7.20	-	-
Kim	1976	[107]	United States	Retrospective cohort	Appendicitis	Not stated	29	75.9	72.60	-	-	66	90
Laborde	2023	[108]	France	Retrospective cohort	CAP	Clinical diagnosis	217	45	-	85.0	-	-	-
Lieberman	1997	[109]	Israel	Prospective cohort	CAP	Clinical diagnosis	145	48.3	-	-	-	-	-
Lim	2001	[110]	United Kingdom	Retrospective cohort	CAP	Clinical diagnosis	156	-	84.10	-	5.39	-	-
Mody	2002	[111]	United States	Retrospective cohort	CAP	Clinical diagnosis	82	2	72.00	-	9.00	-	-
Morrow	1978	[112]	United States	Retrospective cohort	Cholecystitis	Not stated	39	-	70.50	-	-	-	-
Ouriel	1983	[113]	United States	Retrospective cohort	Diverticulitis	Clinical diagnosis	92	38	33.60	-	-	-	-
Pagliano	2015	[114]	Italy	Prospective cohort	Meningitis	Positive microbiology or pathology	131	-	-	-	-	-	-
Parsonage	2009	[115]	United Kingdom	Prospective cohort	CAP	Clinical diagnosis	428	53	-	-	-	-	-
Peled	2006	[116]	Israel	Retrospective cohort	Endocarditis	Not stated	215	-	-	-	-	-	-

Author	Year	Citation	Country	Design	Infection	Infection Definition	Participants	% Female	Age				
									mean	median	sd	lower	upper
Rebelo	2011	[117]	Portugal	Retrospective cohort	Bacteraemia	Positive microbiology or pathology	135	45.9	-	-	-	-	-
Rello	1996	[118]	Spain	Prospective cohort	CAP	Clinical diagnosis	251	26.3	-	-	-	-	-
Salam	2018	[119]	India	Retrospective cross-sectional	Appendicitis	Positive microbiology or pathology	54	44.4	72.00	-	9.30	-	-
Shah	2011	[120]	United States	Retrospective cohort	Diverticulitis	Clinical diagnosis	76	34.2	35.40	-	-	21	40
Shchatsko	2017	[121]	United States	Retrospective cross-sectional	Appendicitis	Positive microbiology or pathology	96	58.3	73.70	-	1.50	-	-
Shirata	2021	[122]	Japan	Prospective cohort	CAP	Clinical diagnosis	872	47.1	82.00	-	8.10	-	-
Starczewski	1988	[123]	United Kingdom	Prospective cohort	CAP	Clinical diagnosis	100	50	82.59	-	7.03	-	-
Storm-Dickerson	2003	[124]	United States	Retrospective cohort	Appendicitis	Clinical diagnostic coding	113	51.3	71.80	-	-	60	98
vanSoest	2022	[125]	Netherlands	Prospective cohort	Meningitis	Positive microbiology or pathology	2140	49.6	-	-	-	-	-
Whitelaw	1992	[126]	South Africa	Prospective cohort	Bacteraemia	Positive microbiology or pathology	121	51.2	74.00	-	-	65	89
Woodford	2009	[127]	United Kingdom	Retrospective cohort	UTI	Clinical diagnostic coding	150	68	84.50	-	-	-	-
Young	2007	[128]	Taiwan	Retrospective cohort	Appendicitis	Clinical diagnostic coding	628	47.8	70.50	-	7.80	-	-
Competence	2008	[129]	Germany	Prospective cohort	CAP	Clinical diagnosis	2647	43.9	-	-	-	-	-
Warmerdam	2017	[130]	Netherlands	Prospective cohort	Infection	Clinical diagnosis	2370	42.4	61.40	-	17.00	-	-
Liu (1)	2024	[131]	China	Retrospective cohort	Sepsis	Not stated	48	33.3	-	71.5	-	-	-
Liu (2)	2024	[131]	China	Retrospective cohort	Sepsis	Not stated	123	43.9	-	76.0	-	-	-
Hunold	2024	[132]	United States	Prospective cohort	CAP	Clinical diagnosis	27	48.1	75.30	-	8.80	-	-
Hsueh (1)	2024	[133]	Taiwan	Retrospective cohort	Bacteraemia	Positive microbiology or pathology	912	44.5	-	79.0	-	-	-
Hsueh (2)	2024	[133]	Taiwan	Retrospective cohort	Bacteraemia	Positive microbiology or pathology	2451	51.9	-	77.0	-	-	-

1. Alpay Y, Aykin N, Korkmaz P *et al*. Urinary tract infections in the geriatric patients. *Pak J Med Sci* 2018;**34**:67–72.
2. Amadoru S, Lim K, Tacey M *et al*. Spinal infections in older people: an analysis of demographics, presenting features, microbiology and outcomes. *Intern Med J* 2017;**47**:182–8.

3. Applefeld MM, Hornick RB. Infective endocarditis in patients over age 60. *Am Heart J* 1974;**88**:90–4.
4. Artero A, Esparcia A, Eiros JM *et al.* Effect of Bacteremia in Elderly Patients With Urinary Tract Infection. *Am J Med Sci* 2016;**352**:267–71.
5. Barsic B, Lisic M, Himbele J *et al.* Pneumococcal meningitis in the elderly. *Neurol Croat* 1992;**41**:131–9.
6. Boonmee P, Ruangsomboon O, Limsuwat C *et al.* Predictors of Mortality in Elderly and Very Elderly Emergency Patients with Sepsis: A Retrospective Study. *West J Emerg Med* 2020;**21**:210–8.
7. Burns JMA, Knight P V. Infective endocarditis in an elderly population: A five-year retrospective study. *Journal of Clinical and Experimental Gerontology* 1991;**13**:161–71.
8. Cabellos C, Verdaguer R, Olmo M *et al.* Community-acquired bacterial meningitis in elderly patients: experience over 30 years. *Medicine* 2009;**88**:115–9.
9. Cataudella E, Giraffa CM, Di Marca S *et al.* Neutrophil-To-Lymphocyte Ratio: An Emerging Marker Predicting Prognosis in Elderly Adults with Community-Acquired Pneumonia. *J Am Geriatr Soc* 2017;**65**, DOI: 10.1111/jgs.14894.
10. Caterino JM, Kline DM, Leininger R *et al.* Nonspecific Symptoms Lack Diagnostic Accuracy for Infection in Older Patients in the Emergency Department. *J Am Geriatr Soc* 2019;**67**:484–92.
11. Caterino JM, Kulchycki LK, Fischer CM *et al.* Risk factors for death in elderly emergency department patients with suspected infection: Clinical investigations. *J Am Geriatr Soc* 2009;**57**:1184–90.
12. Caterino JM, Murden RA, Stevenson KB. Functional status does not predict complicated clinical course in older adults in the emergency department with infection. *J Am Geriatr Soc* 2012;**60**:304–9.
13. Çelikhisar H, Daşdemir Ilkhan G, Arabaci Ç. Prognostic factors in elderly patients admitted to the intensive care unit with community-acquired pneumonia. *Aging Male* 2021;**23**, DOI: 10.1080/13685538.2020.1775192.
14. Chassagne P, Perol MB, Doucet J *et al.* Is presentation of bacteremia in the elderly the same as in younger patients?. *Am J Med* 1996;**100**:65–70.
15. Ciesielski GL. Clinical indicators or urosepsis: a restrospective study of geriatric emergency department admissions. 2010.
16. Covino M, Rosa F, Ojetti V *et al.* Acute Diverticulitis in Elderly Patients: Does Age Really Matter?. *Dig Dis* 2021;**39**:33–41.
17. Domingo P, Pomar V, de Benito N *et al.* The spectrum of acute bacterial meningitis in elderly patients. *BMC Infect Dis* 2013;**13**:108.
18. Dubost J-J, Lopez J, Pereira B *et al.* Primary infectious spondylodiscitis in 51 patients over 75 years old: A comparative study. *Med Clin (Barc)* 2018;**150**:371–5.
19. Elangovan S. Clinical and laboratory findings in acute appendicitis in the elderly. *J Am Board Fam Pract* 1996;**9**:75–8.
20. Erdem H, Coskun O, Kilic S *et al.* Community-acquired acute bacterial meningitis in the elderly in Turkey. *Clinical Microbiology and Infection* 2010;**16**:1223–9.

21. Esposito AL. Community-acquired bacteremic pneumococcal pneumonia. Effect of age on manifestations and outcome. *Arch Intern Med* 1984;**144**:945–8.
22. Ewig S, Klapdor B, Pletz MW *et al.* Nursing-home-acquired pneumonia in Germany: an 8-year prospective multicentre study. *Thorax* 2012;**67**:132–8.
23. Ewig S, Kleinfeld T, Bauer T *et al.* Comparative validation of prognostic rules for community-acquired pneumonia in an elderly population. *Eur Respir J* 1999;**14**:370–5.
24. Fernandez-Sabe N, Carratala J, Roson B *et al.* Community-acquired pneumonia in very elderly patients: causative organisms, clinical characteristics, and outcomes. *Medicine* 2003;**82**:159–69.
25. Finkelstein MS, Petkun WM, Freedman ML *et al.* Pneumococcal bacteremia in adults: age-dependent differences in presentation and in outcome. *J Am Geriatr Soc* 1983;**31**:19–27.
26. Fontanarosa PB, Kaeberlein FJ, Gerson LW *et al.* Difficulty in predicting bacteremia in elderly emergency patients. *Ann Emerg Med* 1992;**21**:842–8.
27. Garcia-Ordonez MA, Garcia-Jimenez JM, Paez F *et al.* Clinical aspects and prognostic factors in elderly patients hospitalised for community-acquired pneumonia. *Eur J Clin Microbiol Infect Dis* 2001;**20**:14–9.
28. Golcuk Y, Golcuk B, Bilge A *et al.* Prognostic value of serum pregnancy-associated plasma protein A level at the initial ED presentation in elderly patients with CAP. *American Journal of Emergency Medicine* 2015;**33**, DOI: 10.1016/j.ajem.2015.05.047.
29. Gopal GK, Wilson BP, Viggeswarpu S *et al.* Clinical Profile and Predictors of Outcomes in Older Inpatients with Pyelonephritis in a Tertiary Care Hospital in Southern India. *J Clin Diagn Res* 2015;**9**:OC05-7.
30. Guirgis FW, Leeuwenburgh C, Grijalva V *et al.* HDL Cholesterol Efflux is Impaired in Older Patients with Early Sepsis: A Subanalysis of a Prospective Pilot Study. *Shock* 2018;**50**:66–70.
31. Hernandez C, Feher C, Soriano A *et al.* Clinical characteristics and outcome of elderly patients with community-onset bacteremia. *J Infect* 2015;**70**:135–43.
32. Horattas MC, Guyton DP, Wu D. A reappraisal of appendicitis in the elderly. *Am J Surg* 1990;**160**:291–3.
33. Horesh N, Shwaartz C, Amiel I *et al.* Diverticulitis: does age matter?. *J Dig Dis* 2016;**17**:313–8.
34. Chou H-L, Han S-T, Yeh C-F *et al.* Systemic inflammatory response syndrome is more associated with bacteremia in elderly patients with suspected sepsis in emergency departments. *Medicine* 2016;**95**:e5634.
35. Jung SK, Rhee DY, Lee WJ *et al.* Neutrophil-to-lymphocyte count ratio is associated with perforated appendicitis in elderly patients of emergency department. *Aging Clin Exp Res* 2017;**29**:529–36.
36. Kaser SA, Furler R, Evequoz DC *et al.* Hyponatremia is a specific marker of perforation in sigmoid diverticulitis or appendicitis in patients older than 50 years. *Gastroenterol Res Pract* 2013;**2013**:462891.

37. Kijsirichareanchai K, Mankongpaisarnrung C, Sutamtewagul G *et al.* Diverticulitis in the young. *J Prim Care Community Health* 2015;**6**:29–34.
38. Kim S-H, Chung J-H, Lee J-C *et al.* Carboxy-terminal provasopressin may predict prognosis in nursing home acquired pneumonia. *Clin Chim Acta* 2013;**421**:226–9.
39. Kim S, Lee K, Kim I *et al.* Red cell distribution width and early mortality in elderly patients with severe sepsis and septic shock. *Clin Exp Emerg Med* 2015;**2**:155–61.
40. Klapdor B, Ewig S, Pletz MW *et al.* Community-acquired pneumonia in younger patients is an entity on its own. *Eur Respir J* 2012;**39**:1156–61.
41. Ko SY, De Groot B, Esteve Cuevas LM *et al.* The association between intravenous fluid resuscitation and mortality in older emergency department patients with suspected infection. *Int J Emerg Med* 2019;**12**:1.
42. Laborde C, Bador J, Hacquin A *et al.* Atypical Presentation of Bacteremic Urinary Tract Infection in Older Patients: Frequency and Prognostic Impact. *Diagnostics (Basel)* 2021;**11**, DOI: https://dx.doi.org/10.3390/diagnostics11030523.
43. Lee C-C, Chen S-Y, Chang I-J *et al.* Comparison of clinical manifestations and outcome of community-acquired bloodstream infections among the oldest old, elderly, and adult patients. *Medicine* 2007;**86**:138–44.
44. Lee JS, Choi HS, Ko YG *et al.* Performance of the Geriatric Nutritional Risk Index in predicting 28-day hospital mortality in older adult patients with sepsis. *Clin Nutr* 2013;**32**:843–8.
45. Lee JS, Jeong KY, Ko SH. Usefulness of the Geriatric Nutritional Risk Index to predict the severity of cholecystitis among older patients in the emergency department. *Geriatr Gerontol Int* 2020;**20**:455–60.
46. Lee WJ, Woo SH, Kim DH *et al.* The neutrophil-to-lymphocyte ratio is associated with bacteremia in older adults visiting the emergency department with urinary tract infections. *Aging Clin Exp Res* 2020;**32**:1129–35.
47. Liu A, Bui T, Van Nguyen H *et al.* Serum C-reactive protein as a biomarker for early detection of bacterial infection in the older patient. *Age Ageing* 2010;**39**:559–65.
48. Luna CM, Palma I, Niederman MS *et al.* The impact of age and comorbidities on the mortality of patients of different age groups admitted with community-acquired pneumonia. *Ann Am Thorac Soc* 2016;**13**, DOI: 10.1513/AnnalsATS.201512-848OC.
49. Ma HM, Tang WH, Woo J. Predictors of in-hospital mortality of older patients admitted for community-acquired pneumonia. *Age Ageing* 2011;**40**, DOI: 10.1093/ageing/afr087.
50. Marinella MA, Mustafa M. Acute diverticulitis in patients 40 years of age and younger. *Am J Emerg Med* 2000;**18**:140–2.
51. Meyers BR, Sherman E, Mendelson MH *et al.* Bloodstream infections in the elderly. *Am J Med* 1989;**86**:379–84.

52. Myint PK, Kamath A V., Vowler SL *et al.* The CURB (confusion, urea, respiratory rate and blood pressure) criteria in community-acquired pneumonia (CAP) in hospitalised elderly patients aged 65 years and over: A prospective observational cohort study [2]. *Age Ageing* 2005;**34**, DOI: 10.1093/ageing/afh234.
53. Mylotte JM, Tayara A, Goodnough S. Epidemiology of bloodstream infection in nursing home residents: evaluation in a large cohort from multiple homes. *Clin Infect Dis* 2002;**35**:1484–90.
54. Parker LJ, Vukov LF, Wollan PC. Emergency department evaluation of geriatric patients with acute cholecystitis. *Acad Emerg Med* 1997;**4**:51–5.
55. Potts FE 4th, Vukov LF. Utility of fever and leukocytosis in acute surgical abdomens in octogenarians and beyond. *J Gerontol A Biol Sci Med Sci* 1999;**54**:M55-8.
56. Rasmussen HH, Sorensen HT, Moller-Petersen J *et al.* Bacterial meningitis in elderly patients: clinical picture and course. *Age Ageing* 1992;**21**:216–20.
57. Raz R, Gersham M, Flatau E *et al.* Acute pyelonephritis in hospitalized women. *Infectious Diseases in Clinical Practice* 1999;**8**:335–40.
58. Riquelme R, Torres A, El-Ebiary M *et al.* Community-acquired pneumonia in the elderly: A multivariate analysis of risk and prognostic factors. *Am J Respir Crit Care Med* 1996;**154**:1450–5.
59. Riquelme R, Torres A, el-Ebiary M *et al.* Community-acquired pneumonia in the elderly. Clinical and nutritional aspects. *Am J Respir Crit Care Med* 1997;**156**:1908–14.
60. Robbins N, DeMaria A, Miller MH. Infective endocarditis in the elderly. *South Med J* 1980;**73**:1335–8.
61. Ruiz LA, Zalacain R, Capelastegui A *et al.* Bacteremic Pneumococcal Pneumonia in Elderly and Very Elderly Patients: Host- and Pathogen-Related Factors, Process of Care, and Outcome. *J Gerontol A Biol Sci Med Sci* 2014;**69**, DOI: 10.1093/gerona/glt288.
62. Ryden CI, Grunditz T, Janzon L. Acute appendicitis in patients above and below 60 years of age. Incidence rate and clinical course. *Acta Chir Scand* 1983;**149**:165–70.
63. Salahuddin O, Malik MAN, Sajid MA *et al.* Acute appendicitis in the elderly; Pakistan Ordnance Factories Hospital, Wah Cantt. experience. *J Pak Med Assoc* 2012;**62**:946–9.
64. Sasaki Y, Komatsu F, Kashima N *et al.* Reactive leukocytosis in older patients with acute colonic diverticulitis: A retrospective study utilizing logistic regression analysis. *Geriatr Gerontol Int* 2020;**20**:951–5.
65. Sheu B-F, Chiu T-F, Chen J-C *et al.* Risk factors associated with perforated appendicitis in elderly patients presenting with signs and symptoms of acute appendicitis. *ANZ J Surg* 2007;**77**:662–6.
66. Shimazui T, Nakada T-A, Oshima T *et al.* Significance of body temperature in elderly patients with sepsis. *Crit Care* 2020;**24**:387.

67. Shimoni Z, Kasem A, Froom P. The influence of mental status on reported local urinary tract symptoms in patients with bacteraemic urinary tract infections. *Int J Clin Pract* 2021;**75**:e13741.
68. Singler K, Bertsch T, Heppner HJ *et al.* Diagnostic accuracy of three different methods of temperature measurement in acutely ill geriatric patients. *Age Ageing* 2013;**42**:740–6.
69. Sirikurnpiboon S, Amornpornchareon S. Factors Associated with Perforated Appendicitis in Elderly Patients in a Tertiary Care Hospital. *Surg Res Pract* 2015;**2015**:847681.
70. Smithson A, Ramos J, Nino E *et al.* Characteristics of febrile urinary tract infections in older male adults. *BMC Geriatr* 2019;**19**:334.
71. Sunden-Cullberg J, Rylance R, Svefors J *et al.* Fever in the Emergency Department Predicts Survival of Patients With Severe Sepsis and Septic Shock Admitted to the ICU. *Crit Care Med* 2017;**45**:591–9.
72. Talebi-Taher M, Javad-Mousavi SA, Arian-Mehr S *et al.* Comparing community acquired pneumonia between elderly population and others. *Iranian Journal of Clinical Infectious Diseases* 2010;**5**.
73. Taniguchi T, Tsuha S, Takayama Y *et al.* Shaking chills and high body temperature predict bacteremia especially among elderly patients. *Springerplus* 2013;**2**:624.
74. Tantarattanapong S, Arwae N. Risk factors associated with perforated acute appendicitis in geriatric emergency patients. *Open Access Emerg Med* 2018;**10**:129–34.
75. Tantarattanapong S, Hemwej T. Door-to-antibiotic Time and In-hospital Mortality of Elder Patients Presenting to Emergency Department with Sepsis; a Cross-Sectional Study. *Arch Acad Emerg Med* 2021;**9**:e44.
76. Terpenning MS, Buggy BP, Kauffman CA. Infective endocarditis: clinical features in young and elderly patients. *Am J Med* 1987;**83**:626–34.
77. Thiem U, Niklaus D, Sehlhoff B *et al.* C-reactive protein, severity of pneumonia and mortality in elderly, hospitalised patients with community-acquired pneumonia. *Age Ageing* 2009;**38**, DOI: 10.1093/ageing/afp164.
78. Tiruvoipati R, Ong K, Gangopadhyay H *et al.* Hypothermia predicts mortality in critically ill elderly patients with sepsis. *BMC Geriatr* 2010;**10**:70.
79. Tsai T-H, Chen K-Y, Yu C-J *et al.* Community-acquired thoracic empyema in older people. *J Am Geriatr Soc* 2005;**53**:1203–9.
80. Valencia AM, Vallejo CE, Alvarez ALL *et al.* Attenuation of the physiological response to infection on adults over 65 years old admitted to the emergency room (ER). *Aging Clin Exp Res* 2017;**29**:847–56.
81. Vincent GM, Amirault JD. Septic arthritis in the elderly. *Clin Orthop Relat Res* 1990:241–5.
82. Warmerdam M, Baris L, van Liebergen M *et al.* The association between systolic blood pressure and in-hospital mortality in older emergency department patients who are hospitalised with a suspected infection. *Emerg Med J* 2018;**35**:619–22.

83. Wasserman M, Levinstein M, Keller E *et al.* Utility of fever, white blood cells, and differential count in predicting bacterial infections in the elderly. *J Am Geriatr Soc* 1989;**37**:537–43.
84. Weisfelt M, van de Beek D, Spanjaard L *et al.* Community-acquired bacterial meningitis in older people. *J Am Geriatr Soc* 2006;**54**:1500–7.
85. Wester AL, Dunlop O, Melby KK *et al.* Age-related differences in symptoms, diagnosis and prognosis of bacteremia. *BMC Infect Dis* 2013;**13**:346.
86. Yahav D, Schlesinger A, Daitch V *et al.* Presentation of infection in older patients--a prospective study. *Ann Med* 2015;**47**:354–8.
87. Yo C-H, Lee M-TG, Hsein Y-C *et al.* Risk factors and outcomes of afebrile bacteremia patients in an emergency department. *Diagn Microbiol Infect Dis* 2016;**86**:455–9.
88. Zhang ZX, Yong Y, Tan WC *et al.* Prognostic factors for mortality due to pneumonia among adults from different age groups in Singapore and mortality predictions based on PSI and CURB-65. *Singapore Med J* 2018;**59**, DOI: 10.11622/smedj.2017079.
89. Andersson A, Bergdahl L. Acute appendicitis in patients over sixty. *American Surgeon* 1978;**44**.
90. Barkham TMS, Martin FC, Eykyn SJ. Delay in the diagnosis of bacteraemic urinary tract infection in elderly patients. *Age Ageing* 1996;**25**, DOI: 10.1093/ageing/25.2.130.
91. Han X, Liu X, Chen L *et al.* Disease burden and prognostic factors for clinical failure in elderly community acquired pneumonia patients. *BMC Infect Dis* 2020;**20**, DOI: 10.1186/s12879-020-05362-3.
92. Chen JH, Chang SS, Liu JJ *et al.* Comparison of clinical characteristics and performance of pneumonia severity score and CURB-65 among younger adults, elderly and very old subjects. *Thorax* 2010;**65**, DOI: 10.1136/thx.2009.129627.
93. Chodak GW, Rangel DM, Passaro E. Colonic diverticulitis in patients under age 40: Need for earlier diagnosis. *The American Journal of Surgery* 1981;**141**, DOI: 10.1016/0002-9610(81)90081-7.
94. Choi JJ, McCarthy MW, Meltzer KK *et al.* The Diagnostic Accuracy Of Procalcitonin for Urinary Tract Infection in Hospitalized Older Adults: a Prospective Study. *J Gen Intern Med* 2022;**37**, DOI: 10.1007/s11606-021-07265-8.
95. Cilloniz C, Ferrer M, Pericàs JM *et al.* Validation of IDSA/ATS Guidelines for ICU Admission in Adults Over 80 Years Old With Community-Acquired Pneumonia. *Arch Bronconeumol* 2023;**59**, DOI: 10.1016/j.arbres.2022.08.012.
96. Conte HA, Chen YT, Mehal W *et al.* A prognostic rule for elderly patients admitted with community-acquired pneumonia. *American Journal of Medicine* 1999;**106**, DOI: 10.1016/S0002-9343(98)00369-6.
97. Cooper C, Cawley MID. Bacterial arthritis in the elderly. *Gerontology* 1986;**32**, DOI: 10.1159/000212794.
98. Cooper GS, Shlaes DM, Salata RA. Intraabdominal infection: Differences in presentation and outcome between younger patients and the elderly. *Clinical Infectious Diseases* 1994;**19**, DOI: 10.1093/clinids/19.1.146.

99. Covino M, Piccioni A, Bonadia N *et al.* Early procalcitonin determination in the emergency department and clinical outcome of community-acquired pneumonia in old and oldest old patients. *Eur J Intern Med* 2020;**79**, DOI: 10.1016/j.ejim.2020.04.055.
100. Gorse G, Thrupp L, Nudleman K *et al.* Bacterial-meningitis in the elderly. *Arch Intern Med* 1984;**144**:1603–7.
101. Habibi S, Babazadeh A, Ebrahimpour S *et al.* Clinical and laboratory findings in elderly with Community-Acquired Pneumonia in Babol, northern Iran - 2017-2019. *Current Issues in Pharmacy and Medical Sciences* 2021;**34**, DOI: 10.2478/cipms-2021-0027.
102. de Groot B, Stolwijk F, Warmerdam M *et al.* The most commonly used disease severity scores are inappropriate for risk stratification of older emergency department sepsis patients: An observational multi-centre study. *Scand J Trauma Resusc Emerg Med* 2017;**25**, DOI: 10.1186/s13049-017-0436-3.
103. Hall A, Wright TM. Acute appendicitis in the geriatric patient. *American Surgeon* 1976;**42**.
104. Hsiao C-Y, Chen T-H, Lee Y-C *et al.* Risk factors for uroseptic shock in hospitalized patients aged over 80 years with urinary tract infection. *Ann Transl Med* 2020;**8**, DOI: 10.21037/atm.2020.03.95.
105. Hui TT, Major KM, Avital I *et al.* Outcome of elderly patients with appendicitis: Effect of computed tomography and laparoscopy. *Archives of Surgery* 2002;**137**.
106. Joo P, Grant L, Ramsay T *et al.* Effect of inpatient antibiotic treatment among older adults with delirium found with a positive urinalysis: a health record review. *BMC Geriatr* 2022;**22**, DOI: 10.1186/s12877-022-03549-8.
107. Kim SK, Smith EB. Acute appendicitis in patients over age 65. *J Natl Med Assoc* 1976;**68**.
108. Laborde C, Deidda M, Bador J *et al.* Apyrexia improves the prognostic value of quick SOFA in older patients with acute pneumonia or bacteremic urinary tract infection. *Infection* 2023;**51**, DOI: 10.1007/s15010-022-01953-1.
109. Lieberman D, Lieberman D, Schlaeffer F *et al.* Community-acquired pneumonia in old age: A prospective study of 91 patients admitted from home. *Age Ageing* 1997;**26**, DOI: 10.1093/ageing/26.2.69.
110. Lim WS, Macfarlane JT. Defining prognostic factors in the elderly with community acquired pneumonia: A case controlled study of patients aged ≥ 75 yrs. *European Respiratory Journal* 2001;**17**, DOI: 10.1183/09031936.01.17202000.
111. Mody L, Sun R, Bradley S. Community-acquired pneumonia in older veterans: Does the pneumonia prognosis index help? *J Am Geriatr Soc* 2002;**50**, DOI: 10.1046/j.1532-5415.2002.50107.x.
112. Morrow DJ, Thompson J, Wilson SE. Acute Cholecystitis in the Elderly: A Surgical Emergency. *Archives of Surgery* 1978;**113**, DOI: 10.1001/archsurg.1978.01370220035006.
113. Ouriel K, Schwartz SI. Diverticular disease in the young patient. *Surg Gynecol Obstet* 1983;**156**.
114. Pagliano P, Attanasio V, Rossi M *et al.* Listeria monocytogenes meningitis in the elderly: Distinctive characteristics of the clinical and laboratory presentation. *Journal of Infection* 2015;**71**, DOI: 10.1016/j.jinf.2015.02.003.

115. Parsonage M, Nathwani D, Davey P *et al.* Evaluation of the performance of CURB-65 with increasing age. *Clinical Microbiology and Infection* 2009;**15**, DOI: 10.1111/j.1469-0691.2009.02908.x.
116. Peled N, Pitlik S, Livni G *et al.* Impact of age on clinical features and outcome of infective endocarditis. *European Journal of Clinical Microbiology and Infectious Diseases* 2006;**25**, DOI: 10.1007/s10096-006-0160-9.
117. Rebelo M, Pereira B, Lima J *et al.* Predictors of in-hospital mortality in elderly patients with bacteraemia admitted to an Internal Medicine Ward. *Int Arch Med* 2011;**4**, DOI: 10.1186/1755-7682-4-33.
118. Rello J. Severe community-acquired pneumonia in the elderly: Epidemiology and prognosis. *Clinical Infectious Diseases* 1996;**23**, DOI: 10.1093/clinids/23.4.723.
119. Salam SS, Chinglensana L, Vanlalremsiama V *et al.* ACUTE APPENDICITIS IN ELDERLY PATIENTS- CHALLENGES IN DIAGNOSIS AND MANAGEMENT. *J Evol Med Dent Sci* 2018;**7**, DOI: 10.14260/jemds/2018/805.
120. Shah AM, Malhotra A, Patel B *et al.* Acute diverticulitis in the young: A 5-year retrospective study of risk factors, clinical presentation and complications. *Colorectal Disease* 2011;**13**, DOI: 10.1111/j.1463-1318.2010.02372.x.
121. Shchatsko A, Brown R, Reid T *et al.* The utility of the alvarado score in the diagnosis of acute appendicitis in the elderly. *American Surgeon*. Vol 83. 2017.
122. Shirata M, Ito I, Ishida T *et al.* Development and validation of a new scoring system for prognostic prediction of community-acquired pneumonia in older adults. *Sci Rep* 2021;**11**, DOI: 10.1038/s41598-021-03440-3.
123. Starczewski AR, Allen SC, Vargas E *et al.* Clinical prognostic indices of fatality in elderly patients admitted to hospital with acute pneumonia. *Age Ageing* 1988;**17**, DOI: 10.1093/ageing/17.3.181.
124. Storm-Dickerson TL, Horattas MC. What have we learned over the past 20 years about appendicitis in the elderly? *Am J Surg* 2003;**185**, DOI: 10.1016/S0002-9610(02)01390-9.
125. van Soest TM, Chekrouni N, van Sorge NM *et al.* Community-acquired bacterial meningitis in patients of 80 years and older. *J Am Geriatr Soc* 2022;**70**, DOI: 10.1111/jgs.17766.
126. Whitelaw DA, Rayner BL, Willcox PA. Community-Acquired Bacteremia in the Elderly: A Prospective Study of 121 Cases. *J Am Geriatr Soc* 1992;**40**, DOI: 10.1111/j.1532-5415.1992.tb04475.x.
127. Woodford HJ, George J. Diagnosis and Management of Urinary Tract Infection in Hospitalized Older People. *J Am Geriatr Soc* 2009;**57**:107–14.
128. Young YR, Chiu TF, Chen JC *et al.* Acute appendicitis in the octogenarians and beyond: A comparison with younger geriatric patients. *American Journal of the Medical Sciences* 2007;**334**, DOI: 10.1097/MAJ.0b013e3180ca8eea.
129. Kothe H, Bauer T, Marre R *et al.* Outcome of community-acquired pneumonia: Influence of age, residence status and antimicrobial treatment. *European Respiratory Journal* 2008;**32**, DOI: 10.1183/09031936.00092507.

130. Warmerdam M, Stolwijk F, Boogert A *et al.* Initial disease severity and quality of care of emergency department sepsis patients who are older or younger than 70 years of age. *PLoS One* 2017;**12**, DOI: 10.1371/journal.pone.0185214.
131. Liu M, Duan Y-J, Zhang Y *et al.* Prognostic Value of Macrophage Inflammatory Protein-3alpha (MIP3-Alpha) and Severity Scores in Elderly Patients with Sepsis. *J Inflamm Res* 2024;**Volume 17**:1503–9.
132. Hunold KM, Mion LC, Gure TR *et al.* Clinical performance of existing diagnostic criteria for pneumonia in older emergency patients: A prospective cohort study. *J Am Geriatr Soc* 2024;**72**:3068–77.
133. Hsueh S-C, Chen P-L, Ho C-Y *et al.* Comparing the Prognostic Impacts of Delayed Administration of Appropriate Antimicrobials in Older Patients with Afebrile and Febrile Community-Onset Bacteremia. *Antibiotics* 2024;**13**:465.

Appendix E: Data Collection

Data was extracted independently by two reviewers using a piloted data collection form. Discrepancies were resolved by discussion and recourse to the original data. Data was collected on:

- The report: author, year, funding status, conflicts of interest
- The study: design (e.g. randomised controlled trial, retrospective cohort), dates, country, infection being studied, the method of identifying patients with infection (e.g. microbiological data from cultures, documented admission diagnosis)
- The participants: inclusion and exclusion criteria, number of participants, baseline demographics (age, sex, ethnicity, severity of illness, co-morbidities)
- The comparator: age groups studied
- The outcome: vital signs studied, measurement method, timepoint studied

For each age group we extracted:

- The number of patients in each group
- The average age of the group (mean or median)
- The age variance of the group (SD, IQR, range)
- The upper and lower limits of the group

Eligible outcomes included heart rate, respiratory rate, temperature, blood pressure and oxygen saturation recorded within 24 hours of hospital admission.

- Where data was presented as a dichotomous or categorical outcome we extracted:
  - The outcome categories
  - The timepoint recorded
  - The number of patients falling within each category and the total number of patients studied
  - The number of missing participants
- Where data was presented as a continuous outcome we extracted:
  - The timepoint recorded
  - The average measure (mean or median)
  - The measure of variance (standard deviation, IQR, range)
  - The number of participants
  - The number of missing participants

Appendix F: Risk of Bias Assessment of Individual Included Studies

High = High risk of bias
Low = Low risk of bias
Unclear = Unclear risk of bias

Author	Year	Citation	Domain			
			1: Patient Selection	2: Index Test(s)	3: Reference Standard	4: Flow and Timing
Shimazui	2020	[66]	Unclear	Unclear	Low	High
Boonmee	2020	[6]	Low	Low	Low	High
Sasaki	2020	[64]	Low	Low	Low	High
Celikhisar	2020	[13]	High	Low	Low	High
Lee	2020	[45]	Low	Low	Low	High
Sunden-Cullberg	2017	[71]	High	High	Low	Low
Hsien-LingChou	2016	[34]	Low	Low	High	High
Caterino	2019	[10]	High	Low	Low	Low
Warmerdam	2018	[82]	High	High	Unclear	High
ZoeXiaozhuZhang	2018	[88]	Low	Low	Low	High
Cataudella	2017	[9]	High	Low	Low	Low
Jung	2017	[35]	High	Low	Low	High
Amadoru	2017	[2]	Unclear	High	Unclear	High
Valencia	2017	[80]	Low	Low	Low	High
Smithson	2019	[70]	High	Low	Low	Unclear
Luna	2016	[48]	Low	Low	Low	High
Golcuk	2015	[28]	High	Low	Low	Low
Lee	2013	[44]	High	Unclear	Low	Unclear
Hernandez	2015	[31]	High	Low	Low	Low
Ruiz	2014	[61]	High	High	Low	Low
Kijsirichareanchai	2015	[37]	Low	High	Low	High
Ma	2011	[49]	High	High	Low	Low
Talebi-Taher	2010	[72]	High	Low	Low	Low
Caterino	2012	[12]	High	High	Low	High
Cabellos	2009	[8]	High	High	Low	Low
Ko	2019	[41]	Unclear	Low	High	High
Weisfelt	2006	[84]	High	High	Low	High
Lee	2007	[43]	High	High	Low	Low
Fernandez-Sabe	2003	[24]	High	High	Low	Low
Thiem	2009	[77]	High	Low	Low	High
Myint	2005	[52]	High	High	Low	Low
Rasmussen	1992	[56]	High	High	Low	High
Potts	1999	[55]	High	High	Low	High
Tsai	2005	[79]	Low	High	Low	High
Ciesielski	2010	[15]	Low	Low	Low	High
Tantarattanapng	2021	[75]	Low	Low	Low	High
Laborde	2021	[42]	Low	Low	Low	High
Shimoni	2021	[67]	High	Low	Low	High
Covino	2021	[16]	Low	Low	Low	High

Author	Year	Citation	Domain			
			1: Patient Selection	2: Index Test(s)	3: Reference Standard	4: Flow and Timing
Lee	2020	[46]	High	High	Low	High
Dubost	2018	[18]	High	High	Low	High
Guirgis	2018	[30]	High	Low	Low	Unclear
Tantarattanapong	2018	[74]	Low	High	Low	High
Alpay	2018	[1]	High	High	Low	High
Artero	2016	[4]	Low	Unclear	Low	High
Yo	2016	[87]	Low	Low	Low	High
Horesh	2016	[33]	High	Low	Unclear	High
Yahav	2015	[86]	High	Low	High	Low
Singler	2013	[68]	High	Low	Low	Low
Wester	2013	[85]	Low	Low	Low	High
Kim	2013	[38]	Unclear	Unclear	Low	Unclear
Domingo	2013	[17]	High	High	Low	High
Kim	2015	[39]	High	Low	Low	High
Gopal	2015	[29]	High	Low	Low	High
Sirikurnpiboon	2015	[69]	Low	High	Low	High
Taniguchi	2013	[73]	High	Low	Low	High
Kaser	2013	[36]	High	High	Low	High
Salahuddin	2012	[63]	High	High	Low	Low
Klapdor	2012	[40]	Unclear	High	Low	Low
Ewig	2012	[22]	High	Low	Low	Low
Tiruvoipati	2010	[78]	Low	Low	Low	High
Liu	2010	[47]	High	Low	Unclear	Low
Erdem	2010	[20]	High	Low	Low	High
Sheu	2007	[65]	High	High	Low	High
Mylotte	2002	[53]	Low	High	Low	High
Garcia-Ordonez	2001	[27]	High	High	Low	High
Marinella	2000	[50]	High	Low	Low	High
Ewig	1999	[23]	High	Low	Low	Low
Parker	1997	[54]	High	Low	Low	High
Riquelme	1996	[58]	High	Low	Low	Low
Elangovan	1996	[19]	High	High	High	High
Chassagne	1996	[14]	High	Low	Low	Low
Fontanarosa	1992	[26]	Low	High	Low	High
Horattas	1990	[32]	High	High	Low	High
Vincent	1990	[81]	High	High	Low	High
Meyers	1989	[51]	High	High	Low	High
Wasserman	1989	[83]	High	Low	Low	Low
Terpenning	1987	[76]	Low	High	Low	High

Author	Year	Citation	Domain			
			1: Patient Selection	2: Index Test(s)	3: Reference Standard	4: Flow and Timing
Esposito	1984	[21]	High	Low	High	Unclear
Finkelstein	1983	[25]	High	High	Low	High
Robbins	1980	[60]	High	Unclear	Low	High
Applefeld	1974	[3]	High	Low	Low	High
Caterino	2009	[11]	High	Low	Unclear	Low
Raz	1999	[57]	High	Low	Low	High
Barsic	1992	[5]	High	Low	Low	High
Burns	1991	[7]	High	High	Low	High
Ryden	1983	[62]	High	High	Low	High
Cilloniz	2023	[95]	Low	Low	Low	Low
Joo	2022	[106]	Unclear	High	Low	High
vanSoest	2022	[125]	High	High	Low	High
Choi	2022	[94]	High	High	Low	Low
Laborde	2023	[108]	High	High	Low	High
Shirata	2021	[122]	High	Low	Low	Low
Habibi	2021	[101]	High	High	Low	High
CAP-ChinaNetwork	2020	[91]	Low	High	Low	High
Covino	2020	[99]	Low	Low	Low	High
Hsiao	2020	[104]	High	Low	Low	High
Salam	2018	[119]	High	Low	Low	High
Warmerdam	2017	[130]	Unclear	Low	Low	Low
deGroot	2017	[102]	Low	Low	High	High
Shchatsko	2017	[121]	High	Low	Low	High
Pagliano	2015	[114]	High	High	Low	Low
Rebelo	2011	[117]	Low	Low	Low	High
Shah	2011	[120]	High	High	Low	High
Chen	2010	[92]	High	High	Low	Low
Parsonage	2009	[115]	High	Low	Low	Low
Woodford	2009	[127]	Low	Low	Low	High
Competence	2008	[129]	High	Low	Low	Low
Young	2007	[128]	High	Low	Low	High
Peled	2006	[116]	High	Low	Low	High
Hui	2002	[105]	High	Low	Low	High
Mody	2002	[111]	High	Low	Low	High
Lim	2001	[110]	High	High	Low	High
Conte	1999	[96]	High	Low	Low	High
Lieberman	1997	[109]	High	High	Low	Low
Rello	1996	[118]	High	High	Low	Low

Author	Year	Citation	Domain			
			1: Patient Selection	2: Index Test(s)	3: Reference Standard	4: Flow and Timing
Barkham	1996	[90]	High	High	Low	High
Cooper	1994	[98]	High	High	Low	High
Whitelaw	1992	[126]	High	Unclear	Low	Low
Starczewski	1988	[123]	High	High	Low	Low
Gorse	1984	[100]	High	High	Low	High
Ouriel	1983	[113]	High	High	Low	High
Chodak	1981	[93]	High	Low	Low	High
Andersson	1978	[89]	High	Low	Low	High
Morrow	1978	[112]	High	High	Low	High
Hall	1976	[103]	High	High	Low	High
Kim	1976	[107]	High	High	Unclear	High
Hunold	2024	[132]	High	Unclear	Low	Low
Hsueh	2024	[133]	Low	Low	Low	High
Liu	2024	[131]	High	Low	Unclear	High
Storm-Dickerson	2003	[124]	High	High	Low	High
Cooper	1986	[97]	High	High	Low	High
